# Employing AlphaFold and TED to explore the structural impacts of stress-associated mutations in rice

**DOI:** 10.1093/bioadv/vbag275

**Published:** 2026-09-22

**Authors:** Fatima Shahid, Neeladri Sen, Christine Orengo, Su Datt Lam

**Affiliations:** Department of Applied Physics, Faculty of Science and Technology, Universiti Kebangsaan Malaysia, 43600 UKM Bangi, Selangor, Malaysia; Department of Structural and Molecular Biology, University College London, London WC1E 7JE, United Kingdom; Department of Structural and Molecular Biology, University College London, London WC1E 7JE, United Kingdom; Department of Applied Physics, Faculty of Science and Technology, Universiti Kebangsaan Malaysia, 43600 UKM Bangi, Selangor, Malaysia; Center for Global Health Research (CGHR), Saveetha Medical College, Saveetha Institute of Medical and Technical Sciences (SIMATS), Saveetha University, Chennai, 602105, India

## Abstract

**Motivation:**

Plants have evolved adaptive mechanisms to handle environmental stress. However, genome-wide association studies (GWASs) have identified genetic variations associated with stress resistance, yet their structural and functional impacts remain poorly understood. This limits our ability to develop more resilient crops.

**Results:**

Using predicted three-dimensional protein structures from the AlphaFold Database, chopped into domains by The Encyclopedia of Domains, we analysed how mutations identified in rice GWASs affect protein structure and function. The analysis revealed 102 mutations in 76 high-quality protein domains likely to impact structure and function. Thirty-nine mutations were positioned within 5 Å of predicted functional sites, including those near post-translational modification sites (3), allosteric sites (5), protein–protein interaction sites (25), ligand-binding sites (15), and conserved regions (9). We identified potential interaction partners for 18 proteins with mutations at predicted interfaces and modelled their complex structures. Six mutations were predicted to enhance binding affinity with their protein partners.

**Availability and implementation:**

This study utilized only open-access tools and and provides a framework that could strengthen plant defence mechanisms. However, experimental validation is essential to confirm the applicability of these findings for plant breeding programs.

## 1 Introduction

Climate change, driven by both natural and human-caused factors, has had a significant impact on global food productivity. Food crops, including rice, maize, and wheat, among others, suffer considerably (Rhaman *et al.* 2022). Notably, half of the world’s population depends mainly on rice (*Oryza sativa*), which is cultivated on roughly 160 million hectares. It is a staple food throughout Asia, where it feeds more than 46% of the population (Shah 2021). Rice is a diploid species with a genome size of approximately 430 Mb, organized into 12 chromosomes. The reference genome contains roughly 40 000 protein-coding genes, making it a well-characterized model for cereal proteomics studies (Sasaki and Burr 2000). However, rice productivity is threatened by climate-induced abiotic stresses (ASs), such as heat, drought, and salinity (Dar *et al.* 2021). Rice yield is also significantly decreased by biotic stress (BS) factors such as insects and pests (e.g. the brown planthopper, which feeds on phloem sap) (Li *et al.* 2021). With the intensification of climate change, these stresses are projected to reduce global rice yields by 10–25% by 2050, creating an urgent need for stress-tolerant varieties to ensure food security for billions of people (Teng *et al.* 2016, Aswathi *et al.* 2022).

In response to stress challenges, plants have evolved sophisticated defence mechanisms (Rane *et al.* 2021, Kaur *et al.* 2022). For instance, constitutive defence structures such as cell walls, waxy cuticles, and bark serve as the first line of defence (Mageroy *et al.* 2023). In addition, plants have inducible mechanisms, including PAMP-triggered immunity (PTI) and effector-triggered immunity (ETI), intricate signalling cascades, the generation of reactive oxygen species (ROS), hormone activation, and the synthesis of secondary metabolites (SMs) such as phytoalexins and phenolics (Kumar *et al.* 2023). A plethora of plant proteins, such as catalytic enzymes, transcription factors, and receptors, along with metabolites, play pivotal roles in combating stress (Kumar *et al.* 2020, Suman *et al.* 2021, Yaqoob *et al.* 2022). In Arabidopsis (commonly known as mouse-ear cress), the High-affinity Potassium Transporter (HKT1) helps remove sodium from the cell during salt stress. A single amino acid substitution at residue 207, from asparagine (ASN) to aspartic acid (ASP), has been found to augment salt tolerance. This substitution has been experimentally proven to increase sodium export and enhance potassium uptake, thereby helping the plant better withstand high-salinity conditions (Ali *et al.* 2016). Further exploration of stress-resistance strategies through proteomics and metabolomics is essential to elucidate and enhance the resilience of these vital crops, thereby securing global food production (Jha *et al.* 2022, Shahid *et al.* 2025).

Towards this aim, researchers have used genome-wide association study (GWAS) datasets to study mutations in the plant genomes that are putatively responsible for stress mediation. However, pinpointing the most impactful mutations has been challenging due to their sheer number (Gundersen 2014, Duncan *et al.* 2019). Previous analyses have identified GWAS associations without systematically evaluating the structural and functional consequences of any detected mutations (Pearce and Zhang 2021). However, with the establishment of the AlphaFold Protein Structure Database (AFDB) at the European Bioinformatics Institute, which comprises 214 million predicted protein structures, with ∼19 million belonging to *Viridiplantae* (green plants), and The Encyclopedia of Domains (TED) (Lau *et al.* 2024), which accurately segments protein structures into their constituent domains, we can now use structural data for these types of studies (Jumper *et al.* 2021, Abramson *et al.* 2024, Lau *et al.* 2024, Shahid *et al.* 2025). Domain regions in TED have been mapped to CATH superfamilies, which allows for the detection of conserved residues that may be implicated in protein function and the inheritance of known functional sites from other domains within the same family. Mutations that lie near these sites may have functional impacts.

In this study, we used high-quality rice structures from AFDB segmented into domains via TED to predict the impacts of residue mutations linked to stress resistance. We used the GWAS Atlas, a comprehensive, manually curated knowledge base developed by Liu *et al.* that integrates genome-wide genotype-to-phenotype associations across diverse species (Liu *et al.* 2023). The identified missense variants were linked to stress control by mapping them onto genes that, within this resource, are reported to carry genome-wide significant associations with BS and AS tolerance traits in rice and related plant species. Each candidate was therefore prioritized on the basis of these curated, statistically supported genotype–phenotype associations, rather than on sequence-based functional prediction alone. By identifying mutations that may have a functional impact, this study may aid the engineering of more resilient stress-tolerant rice strains. However, experimental validation would be required to verify the predicted effects.

## 2 Methods

### 2.1 Retrieval of good quality structural domains from TED

We obtained AlphaFold [v.4, (Jumper *et al.* 2021)] predicted protein domain structures from the TED resource (Lau *et al.* 2024) (on 4 October 2024) belonging to *O. sativa* subsp. *japonica* (Asian rice). TED (https://ted.cathdb.info) comprises around 365 million potential domains, identified from ∼214 million proteins, with ∼19 million proteins belonging to green plants (*Viridiplantae*) (Lau *et al.* 2024). TED uses a consensus approach comprising three independent methods to identify domain boundaries: Chainsaw, Merizo, and UniDoc (Lau *et al.* 2023, Wells *et al.* 2023, Zhu *et al.* 2023). It also employs structure comparison tools, such as Foldseek and other deep learning methods (Merizo-search), to assign these domains to CATH domain superfamilies (Van Kempen *et al.* 2024). Full details of the TED protocol can be found in Lau *et al.* (2024).

Good-quality TED domain structures for each plant protein were extracted from TED based on the following metrics, i.e. domains with (1) predicted local distance difference test (pLDDT) score ≥70, (2) ≥ 6 secondary structural elements (SSEs), (3) normalized radius of gyration (nrg) < 0.356, as defined by Bordin *et al.* (2023), (4) packing density > 10, according to Bordin *et al.* (2023), and also (5) *Q*-score > 70, a confidence metric that reflects the predicted quality and reliability of the protein structure model (Jumper *et al.* 2021). Notably, local model uncertainty directly impacts prediction reliability; we therefore excluded these regions from analysis and repeated enrichment calculations stratified by pLDDT threshold to confirm robustness. The normalized radius of gyration reports the mass distribution of a domain relative to that expected for a compact sphere of equivalent volume, thereby providing a size-independent descriptor of global shape; low values indicate a spherical, compact fold, whereas elevated values reflect elongated or partially extended conformations (Bordin *et al.* 2023). Moreover, the packing density quantifies the mean number of residue neighbours within a fixed distance cut-off, serving as a proxy for the density of the hydrophobic core; well-folded domains with tightly interdigitated interiors yield high values, while structures containing internal cavities or lacking a buried core yield low values. The threshold enforces the presence of a solid, contact-rich interior that global-shape measures alone cannot verify. Lastly, the *Q*-score is a composite quality metric derived as a weighted combination of structural alignment coverage against CATH representatives, the consensus level across independent domain-parsing methods, domain globularity, and the normalized mean pLDDT; it thus reflects the overall confidence in a domain assignment rather than a single geometric property. The threshold retains only annotations supported concordantly across these criteria (Lau *et al.* 2024).

### 2.2 GWAS data acquisition

The GWAS Atlas was used to extract missense mutations, likely to confer stress tolerance in rice (Liu *et al.* 2023). The current release of the GWAS Atlas includes 163 479 genotype-to-phenotype associations for 461 traits, derived from 219 rice-specific publications. The GWAS Atlas is a valuable resource used for agronomic trait studies, crop selection and protein engineering. This database provides annotations from multiple ontologies for filtering the data, including biochemical processes, plant morphology, stress responses, and others. For this study, the plant stress category (both abiotic and biotic) was selected from the Plant Phenotype Ontology and Trait Ontology section. Since GWAS datasets provide only nucleotide data for variants, information on the amino acid substitutions in the proteins was acquired using Genome Variation Map (Song *et al.* 2018), a web server that provides variant annotation including Gene ID, Transcript ID, predicted variant impact of the genetic variant on the phenotype, allele change (mutations), protein ID, amino acid residue number where the change occurs, as well as residue change (Li *et al.* 2021). These mutations were further filtered to retain missense mutations. UniProt IDs were used to retrieve protein sequences from UniProt, and missense mutations were then mapped to high-quality TED structures.

### 2.3 Prediction of the impact of mutations

Changes in physicochemical properties of the mutations were analysed for their effect based on the Grantham score (GS). The Grantham metric quantifies changes in physicochemical properties (Grantham 1974). Mutations were labelled as radical (>150), moderately radical (100–150), moderately conservative (51-100) and conservative (<50) based on their GS ranges. A subset of mutations was selected based on GWAS missense mutations with GSs surpassing 50 that mapped to good-quality TED structures (26). The impact of the mutations on the protein was also assessed using MutPred2 (a machine-learning tool that predicts the effects of amino acid substitutions on protein structure and function, with ≥ 0.5 indicating impactful mutations). However, we did not discard any mutation-containing domains based on this analysis, as the tool has not yet been benchmarked for plants (Pejaver *et al.* 2020).

### 2.4 Annotation of mutation-containing domains

The mutation-containing domains were annotated using the PANTHER and CATH superfamily resources (Mi *et al.* 2021, Sillitoe *et al.* 2021). We also examined annotations from Pfam and other InterPro resources (Blum *et al.* 2021, Mistry *et al.* 2021). Subsequently, we performed functional enrichment studies on the proteins having mutations using g:Profiler with default parameters (Kolberg *et al.* 2023). The proteins with mutation-containing domains were also annotated through trait analysis. This step used the published literature associated with the GWAS Atlas to understand the phenotypic trait impacted by a specific mutation.

### 2.5 Prediction of functional sites and minimal distance calculation

Since our dataset of stress resistance-associated mutations included proteins with sparse functional site annotations, various tools were used to predict functional residues. P2Rank (Krivák and Hoksza 2018) was used to predict ligand-binding pockets, setting a probability cut-off of 0.5. We also used the AlphaFill webserver to detect likely ligand-binding sites, only selecting matches with medium to high confidence and local RMSD below 1 Å (a lower local RMSD indicates high structural similarity and therefore more optimal ligand transfer) (Hekkelman *et al.* 2023). Because both AlphaFill and P2Rank predict ligand-binding sites, all predicted sites were treated as small-molecule binding sites; overlapping predictions were merged into a single site and non-overlapping ones were counted separately. For analysing protein–protein interaction sites, Cons-PPISP was employed, identifying residues with >50% probability (Jordan *et al.* 2012, Krivák and Hoksza 2018). Allosteric sites were predicted using PASSer 2.0, retaining those with a confidence score exceeding 50% (Tian *et al.* 2023). Post-translational modification (PTM) sites were predicted using PDBSiteScan, which searches three-dimensional (3D) protein fragments structurally similar to known site templates from the PDBSite collection. This identification relies on finding sites where the maximum distance mismatch between the N, Cα, and C atoms of the protein fragment and the template is less than 2.0 Å (Ivanisenko *et al.* 2004).

Conserved residues were predicted using an in-house protocol (Das *et al.* 2015). Homologues were identified using PSI-BLAST (Altschul *et al.* 1997), with an *E*-value threshold of 0.005 against the non-redundant UniProt database. This was followed by multiple sequence alignment (MSA) using ClustalOmega (Altschul *et al.* 2009, Sievers and Higgins 2021). For MSAs with sufficiently diverse sequences [diversity of positions score (DOPS) > 70%], highly conserved residues were identified with a ScoreCons value greater than or equal to 0.7 (Sedaghatinia *et al.* 2009, Yin *et al.* 2025). Highly conserved positions are likely to be of functional importance, e.g. catalytic sites or substrate binding sites (Das *et al.* 2015). Domains possessing predicted or known functional sites were manually analysed using UCSF Chimera (Wang and Samudrala 2006).

The minimal distance between mutated residues and predicted functional site residues was calculated. Only mutations ≤5 Å distance from a predicted functional site were considered for further examination.

### 2.6 Stability check

For domains where mutations were >5 Å distance from a functional site, the effect of the mutation on the stability of the protein structure was evaluated using FoldX (Buß *et al.* 2018). The ΔΔ*G* value was determined, representing the difference in energy between the mutant and wild-type protein. A negative value designates a stabilizing mutation while a positive value indicates a destabilizing mutation (Buß *et al.* 2018). We used a criterion of ≥0.5 kcal/mol to highlight significant change.

### 2.7 Validation analyses

We checked our analysis using a random model to examine whether the observed GSs of all the stress-associated mutations mapped to high-quality TED domain structures differed from those expected by chance. For each stress-associated mutation, we randomly selected a residue within the same domain and assigned an alternative amino acid chosen from the 19 remaining standard amino acids. The GS for each resulting substitution was then calculated. This process was repeated 10 000 times to establish the expected background distribution. We then calculated the proportion of random substitutions falling into each GS category and compared it with the observed distribution.

We also checked our analysis using random models to examine the proximity of randomly selected residues to functional sites and calculate statistical significance. For every mutated residue, we randomly selected residues within the same domains 10 000 times and determined the percentage of residues that lie close to functional sites. This was done by randomly selecting a residue and then identifying residues that are close (<5 Å) to the selected residue. The overall percentage for all random events was then computed for each functional site. This percentage  was then compared with the observed mutation percentage. The statistical significance was assessed using enrichment-based hypothesis testing, followed by Benjamini–Hochberg correction for multiple comparisons. All analysed functional categories showed significant enrichment after false discovery rate (FDR) correction (*q* < .05), demonstrating that the observed mutations are non-randomly distributed and preferentially localize to biologically relevant regions rather than reflecting protein architecture or accessibility biases alone. To validate our protein stability analysis across a larger number of mutations, we repeated the stability analysis for the same random mutation set (10 000×) and recorded the randomly found and original percentages.

The 5 Å distance threshold was selected based on established structural biology conventions, as this distance encompasses typical hydrogen bonding interactions (2.5–3.5 Å) and van der Waals contacts (∼4 Å), representing biologically relevant spatial relationships (Ferreira de Freitas and Schapira 2017, Kurkcuoglu *et al.* 2018). To assess the robustness of this choice, we performed a sensitivity analysis across multiple distance thresholds (3–8 Å) and reported the changing trends in functional site count.

Lastly, for variants without known functional annotations, we searched for similar protein structures in the RCSB database (identity ≥25%, *E*-value ≤0.01, ≤10 templates per protein) and aligned them to the corresponding query domain. These homologous structures were then examined for two types of evidence that may indicate functional importance. First, we identified residues located close to bound DNA or RNA molecules (within 5 Å) in nucleic-acid-bound homologous structures, suggesting a potential role in nucleic acid binding. Second, we analysed water molecules consistently observed in the same structural position across multiple homologs. Water positions present in at least 60% of homologous structures were considered conserved (1.5 Å clustering radius, conservation score = fraction of templates showing a water at that position) and may indicate structurally or functionally important sites.

### 2.8 Protein–protein interaction analysis

For proteins that were predicted near protein–protein interaction sites (Section 2.5), potential interacting protein partners were identified by screening the STRING database version 12.0 and an extensive literature survey (Szklarczyk *et al.* 2023). For the STRING tool, a confidence cut-off of >0.5 was used to consider high-confidence protein–protein interactions (Taboada *et al.* 2010, Zhu *et al.* 2016). For predicted interactors, AlphaFold 3 was used to model the protein complex (Abramson *et al.* 2024). These were screened for their pLDDT (≥70), predicted template modelling (pTM) score (≥0.5), and interface predicted TM score (ipTM) (≥0.6). Further quality evaluations were performed using PIZSA, a method that assesses the likelihood of protein-protein interactions based on their 3D structures, in which a *Z*-score above 1 indicates a stable interface (Roy *et al.* 2019). Predicted changes in protein–protein binding affinity upon mutation were calculated using mCSM-PPI2. Following the convention of Rodrigues *et al*., positive mCSM-PPI2 ΔΔ*G* affinity values indicate increased binding affinity, whereas negative values indicate decreased binding affinity. Variants with mCSM-PPI2 ΔΔ*G* affinity >0.5 kcal/mol were considered to show a substantial increase in binding affinity (Xue *et al.* 2016, Rodrigues *et al.* 2019).

## 3 Results

### 3.1 Model quality assessment of domains

We extracted 99 395 AlphaFold predicted domain structures for *O. sativa* subsp. *Japonica* from TED that had good pLDDT values (≥70) and good packing density and globularity (see Section 2 for further details). Of these, 77 787 domains (∼78%) were classified in CATH superfamilies/folds by the TED assignment protocol [please see Lau *et al.* (2024)]. This is a significant increase (200-fold) over the 360 experimental PDB structures for rice in the CATH domain superfamilies [v 4.3 (Bordin *et al.* 2023)] (Table 1, available as supplementary data at *Bioinformatics Advances* online).

**Table 1:** Phenotypic trait annotation of mutations within protein domains (as categorized by the GWAS Atlas.

Traits	# of selected mutations	# of domains	Reference (s)
**Rice bacterial blight disease resistance**	37	25	Jiang *et al.* (2021), Lu *et al.* (2021), Yang *et al.* (2021), Wang *et al.* (2024)
**Brown planthopper resistance**	21	11	Zhou *et al.* (2021)
**Sheath blight disease resistance**	15	13	Zhang *et al.* (2019)
**Rice yellow mottle virus resistance**	9	9	Cubry *et al.* (2020)
**Anaerobic stress response index**	5	5	Hsu and Tung (2015)
**Altered seedling numbers due to salt stress**	4	3	Liu *et al.* (2019)
**Total lesion length induced by sheath blight**	3	3	Zhang *et al.* (2019), Thesiya *et al.* (2023)
**Seedling survival days associated to salinity tolerance**	3	2	Naveed *et al.* (2018)
**Cold tolerance**	1	1	Wang *et al.* (2016)
**Nitrogen-induced susceptibility to rice blast fungus**	2	2	Frontini *et al.* (2021), Hui *et al.* (2024)
**Relative salt affected root surface area**	1	1	Wang *et al.* (2022)
**Blast disease resistance**	1	1	Volante *et al.* (2020)

### 3.2 GWAS analysis

A total of 3034 missense variations in rice proteins were extracted from the GWAS Atlas (Liu *et al.* 2023). Out of these, 709 mutations were present in stress-associated proteins. Among them, 172 mutations were mapped to 113 high-quality domain structures from TED (from 101 proteins). Based on Grantham score (GS) classification, nine mutations were radical changes (≥150 GS), 28 were moderately radical (100–150 GS), 65 were moderately conservative (50–100 GS), and 70 were conservative (<50 GS) with respect to the physicochemical properties of the substituted residue. The 70 conservative mutations were then excluded from our subsequent analysis.

To check whether this pattern of more conservative and moderately conservative mutations, as opposed to radical and moderately radical ones, is typical for GWAS-identified missense variants, we analysed GWAS-identified mutations from other crops (maize and soybean) in the GWAS Atlas (Liu *et al.* 2023). We downloaded 121 and 30 missense mutations from maize and soybean, of which 25 and 18 were associated with stress-related signalling. Upon mapping to good-quality domains, 23 (19 domains, 18 proteins) and five (five proteins, five domains) mutations were available for analysis. We observed a similar distribution of GS categories in maize and soybean, with conservative and moderately conservative mutations occurring more than radical and moderately radical ones. While sample sizes for maize and soybean are limited, the consistent patterns across three crop species suggest that this distribution may not be specific to rice. Please refer to Table 2a, available as supplementary data at *Bioinformatics Advances* online for more information.

**Table 2:** Mutations near functional sites.

# mutations that lie within 5 Å of					# unique mutations that lie within 5 Å of a functional site
PTM site	Allosteric site	PPI site	Ligand-binding site	Conserved site	
**3**	5	25	15	9	39

We further checked whether this distribution occurred by chance by running 10,000 random variants in the same proteins. We generated a random model in which 172 mutations were repeatedly sampled (10 000 iterations) from the same set of 101 proteins, and their GSs were calculated to establish an expected background distribution.

Stress-associated mutations showed a distinct GS distribution compared with the random model. Conservative substitutions were more than twice as frequent in stress-associated mutations (40.7%) as in random mutations (17.9%). The proportion of moderately conservative substitutions was broadly similar between the two groups (37.8% versus 36.9%). In contrast, disruptive substitutions were less common. Moderately radical changes accounted for 16.3% of stress-associated mutations compared with 29.0% in the random model, while radical changes made up only 5.2% compared with 16.2%. Please refer to Table 2b, available as supplementary data at *Bioinformatics Advances* online.

MutPred2 also predicted that 37% (38/102) were likely to have a significantly impact. However, it should be noted that MutPred2 has not yet been benchmarked for plant species (Table 1, available as supplementary data at *Bioinformatics Advances* online). For 37 variants with GSs above 100 (classified as moderately radical or radical changes), only 13 also had MutPred2 scores above 0.5 (35% of cases).

#### 3.2.1 GWAS trait analysis

Out of a total of 102 rice mutations analysed in 76 good-quality domains, 88 mutations (in 64 domains) from studies linked to the GWAS Atlas were found to be associated with biotic stress (BS). Resistance to rice bacterial blight disease caused by the pathogen *Xanthomonas oryzae* is associated with 37 mutations across 25 domains. Fifteen mutations (13 domains) were associated with resistance to Sheath Blight Disease (*Rhizoctonia solani*) (Zhang *et al.* 2019, Wang *et al.* 2021). Nine and 21 mutations (in nine and 11 domains, respectively) were associated with yellow mottle virus resistance and brown planthopper (*Nilaparvata lugens*) resistance, respectively. The six remaining mutations in six domains related to BS were associated with different traits that result from stress mitigation strategies employed by the plant [blast fungus susceptibility (Frontini *et al.* 2021), blast disease resistance (Volante *et al.* 2020), and total lesion length induced by sheath blight (Zhang *et al.* 2019)].

The remaining 14 mutations (out of the total 102) in 12 domains have been reported in other studies to be related to abiotic stresses (ASs) (Lafarge *et al.* 2017, Li *et al.* 2017, Kumar *et al.* 2021, Le *et al.* 2021) (see Table 1 and Supplementary S1, available as supplementary data at *Bioinformatics Advances* online).

### 3.3 Functional annotations

#### 3.3.1 PANTHER annotations

We were able to assign 55 domains in 54 proteins (out of 74 analysed proteins) with 73 mutations, distributed across 46 families/subfamilies, with the majority representing stress-response protein families. The most abundant protein classes included antimicrobial response proteins, mostly containing NB-ARC domains, as well as transmembrane signal receptors, proteases, and protease inhibitors, collectively indicating active proteolytic regulation during stress responses (see File S3, available as supplementary data at *Bioinformatics Advances* online for details).

#### 3.3.2 CATH annotations

Of the 76 domain structures analysed, 64 domains in 63 proteins, harbouring 81 out of 102 mutations, were mapped to 33 distinct CATH superfamilies. Another 11 domains in 10 proteins, having 21 mutations, were classified only at the fold level (eight different folds) in the TED dataset (File S4 and b, available as supplementary data at *Bioinformatics Advances* online).

While most domains harbouring mutations were classified within superfamilies of essential enzymes and housekeeping proteins, such as transferases (1.10.510.10), a notable portion, 19 out of the mapped 81 mutations, occurred in superfamilies known to modulate stress responses. Specifically, there were nine mutations in the superfamily 3.80.10.10 (ribonuclease inhibitor) and 10 mutations in the superfamily 3.40.50.300 [P-loop containing nucleotide triphosphate hydrolases (NTPases)]. Proteins belonging to these superfamilies have been associated with different abiotic and biotic stress mediation. For instance, three proteins with four domains and nine mutations (Q0IR17_30_39_53_154, Q0IR17_45_199, A0A0N7KU10_645_768_784_806, and Q2R078_99_147_165_422) from our set have copies of the ribonuclease inhibitor (RI) domain characterized by leucine-rich repeats that are crucial for pathogen recognition (Holt *et al.* 2000). The P-loop-containing NTPase OsGAP1 has been found to play a role in regulating plant responses to salt stress (Cheung *et al.* 2013). P-loop NTPase domains function by hydrolyzing energy-containing molecules, e.g. ATP or GTP, to drive various cellular processes. In wheat, they are involved in activating the resistance pathway against diseases caused by stripe rust and powdery mildew (Zhang *et al.* 2022).

#### 3.3.3 Pfam and InterPro annotations

We searched for further annotations in Pfam and InterPro. Sixty-six out of the 76 domains had InterPro annotations, representing 86.8%. Sixty-six out of the 76 domains had Pfam domain annotations. Sixty-three out of the 66 domains (95.45%) had similar annotations from both systems. Both resources identified proteins with stress-related domains, including NB-ARC domains, leucine-rich repeat domains and chitinases (File S5, available as supplementary data at *Bioinformatics Advances* online).

#### 3.3.4 g:Profiler functional enrichment study

Functional enrichment analysis by g:Profiler revealed that our protein-coding gene set was strongly enriched for plant defence mechanisms centred on cell wall degradation pathways. Fifty-one genes (69% of the total) could be mapped to pathways that showed significant enrichment for processes involved in breaking down cell wall polysaccharides, with four genes (7.8%) involved in xylan and hemicellulose catabolism (*P* = 3.8 × 10^−6^), and three genes (5.9%) showing chitinase activity that targets fungal cell walls. The molecular functions emphasize catalytic activities, with 26 genes (51%) showing general catalytic activity and four genes (7.8%) having hydrolase activity that degrades carbohydrate bonds. This pattern, combined with significant enrichment of eight genes (15.7%) in defence response pathways and six genes (11.8%) responding to biotic stimuli, suggests our gene set represents a coordinated plant immune response that uses enzymatic degradation to break down pathogen cell walls, a mechanism where the plant secretes enzymes to digest invading microorganisms, particularly fungi. The extracellular localization of eight genes (15.7%, *P* = .0003) further supports this interpretation, as these defensive enzymes need to be secreted outside the cell to encounter and neutralize pathogens in the apoplastic space (File S6, available as supplementary data at *Bioinformatics Advances* online).

The functional annotation analyses reveal that our dataset comprises proteins largely associated with plant stress response and defence mechanisms, as supported by PANTHER, CATH, Pfam, InterPro, and g:Profiler databases. PANTHER annotations identified 72% of proteins as belonging to stress-response families, with antimicrobial response proteins containing NB-ARC domains being most abundant (accounting for 18.5% of the annotated PANTHER class category), together with transmembrane receptors and proteases involved in stress regulation. CATH analysis reinforced this pattern, finding that 18 mutations occurred in domains from stress-response superfamilies (22%), particularly RIs with leucine-rich repeats crucial for pathogen recognition, and P-loop NTPases involved in disease resistance pathways. Pfam and InterPro annotations showed high concordance (95.45%) in identifying stress-related domains, including NB-ARC domains, leucine-rich repeats, and chitinases, across 86.8% of domains. The g:Profiler functional enrichment analysis provided the most specific mechanistic insight, revealing that 51% of genes have catalytic activity focused on cell wall degradation, with significant enrichment for chitinase activity (5.9% of genes) and polysaccharide breakdown (7.8% for xylan/hemicellulose catabolism), alongside 15.7% of genes involved in defence responses and extracellular localization.

Together, these five complementary annotation resources paint a coherent picture of a gene set associated with plant immune responses that recognize pathogens through leucine-rich repeat domains and combat them through secreted enzymes that degrade pathogen cell walls, representing a ‘detect and destroy’ defence system.

### 3.4 Proximity of mutations to functional sites and protein–protein interactions

We predicted various functional sites for the proteins in our protein dataset, such as PTMs, allosteric sites, PPI sites, ligand-binding sites, and conserved sites (see Section 2 for details). Thirty-nine (out of 102) mutated residues are close (i.e. ≤5 Å) to predicted functional sites (see Table 2 and Fig. 1). Detailed information is present in File S7, available as supplementary data at *Bioinformatics Advances* online. We also found that 15 out of these 39 mutations are close to at least two predicted functional sites.

**Figure 1:**
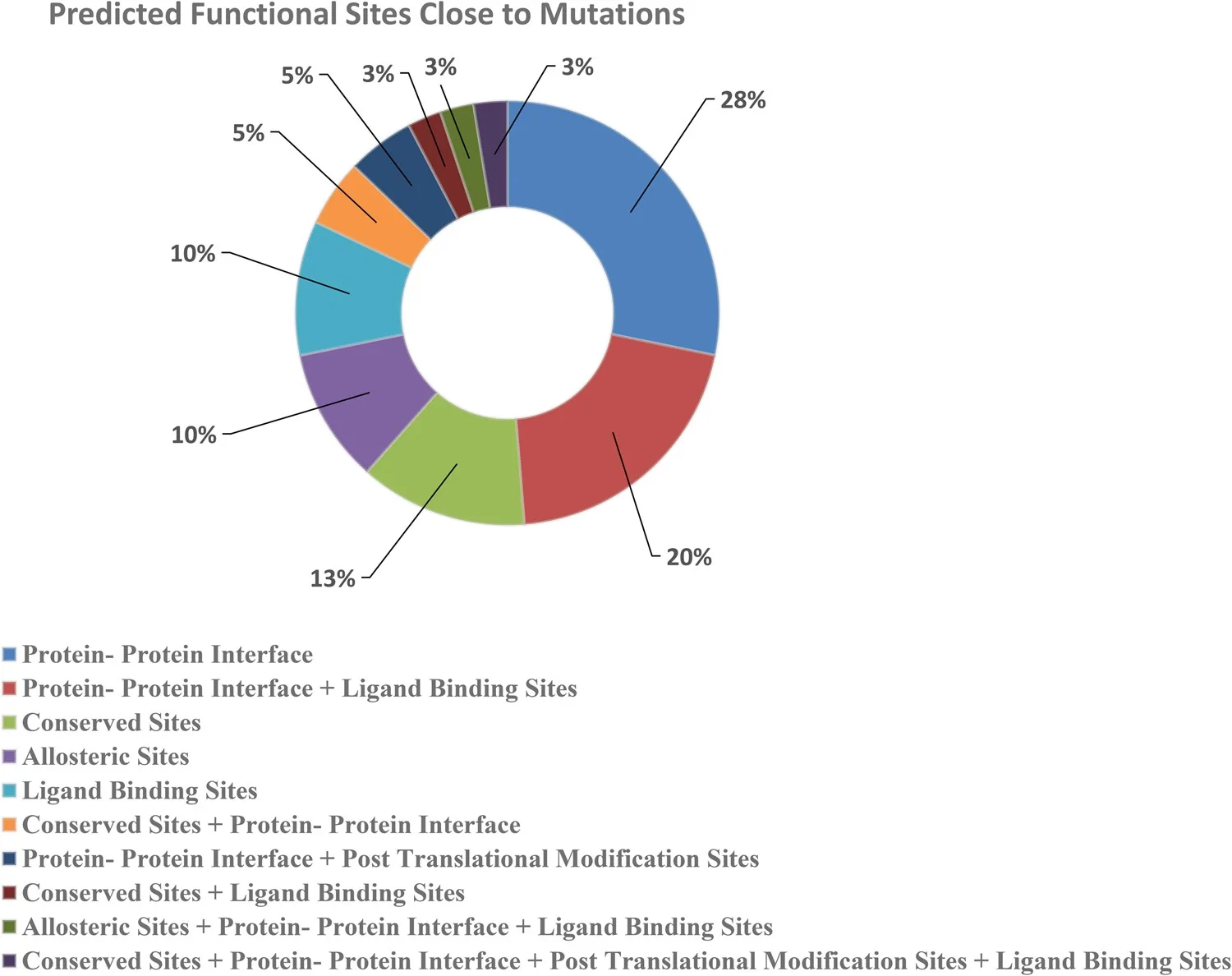
Distribution of mutations near protein functional sites. A total of 39 mutations were analysed and categorized by their proximity to single or co-occurring functional sites (i.e. sites having multiple functional site annotations). Most mutations were found near single sites (24), while a smaller subset was located at the intersection of two (13), three (1), or four (1) distinct functional sites.

Further analyses revealed that out of these 39 mutations mapped to 34 domains, 34 mutations belong to 30 domains mapped to 20 CATH superfamilies. The remaining five mutations in four domains could be mapped to four CATH fold groups.

Twenty-five mutations (belonging to 23 domains from 23 proteins) out of 39 missense mutations lie close to predicted protein–protein interaction sites. We identified their putative protein partners using the STRING database and an extensive literature survey. Eighteen high-confidence interactions with a STRING score above 0.5 and strong biological support from the literature (File S8, available as supplementary data at *Bioinformatics Advances* online) were modelled using AlphaFold 3.

Out of these 18 models, 10 models had a pTM >0.5 and an ipTM score >0.6, reflecting high-quality models. Six out of these 10 complexes also had a PRODIGY value < −7 kcal/mol and a PIZSA *Z*-score above 1, showing good-quality interactions. We ran mCSM-PPI2 for the six complexes and found that these showed an increase in the affinity between the protein domain and the predicted binder for the mutated residue. For the selected examples, we used the confidence cut-offs of mCSM-PPI2 ΔΔ*G* affinity ≤ −0.5 kcal/mol (for destabilizing; affinity-reducing) and ≥0.5 kcal/mol (for stabilizing; affinity-enhancing), to analyse the structural impact.

To test whether mutations cluster near functional sites more than expected by chance, we compared observed mutations to randomly selected ones. This approach allows us to determine whether the occurrence of mutation clusters near functional sites exceeds what would occur randomly. Statistical significance was assessed using enrichment-based hypothesis testing, followed by Benjamini–Hochberg correction for multiple comparisons. All analysed functional categories showed significant enrichment after FDR correction (*q* < .05), demonstrating that the observed mutations are non-randomly distributed and preferentially localize to biologically relevant regions rather than reflecting protein architecture or accessibility biases alone (Table 9a, available as supplementary data at *Bioinformatics Advances* online).

Detailed enrichment statistics, including observed and expected mutation frequencies, fold enrichment values, raw *P*-values, FDR-adjusted *q*-values, and significance assessments for each functional category, are summarized in Table 9a, available as supplementary data at *Bioinformatics Advances* online and Fig. 2. Protein–protein interface sites showed the strongest statistical support, followed by ligand-binding, PTM-associated, allosteric, and conserved regions, highlighting a significant accumulation of mutations within functional protein regions.

**Figure 2:**
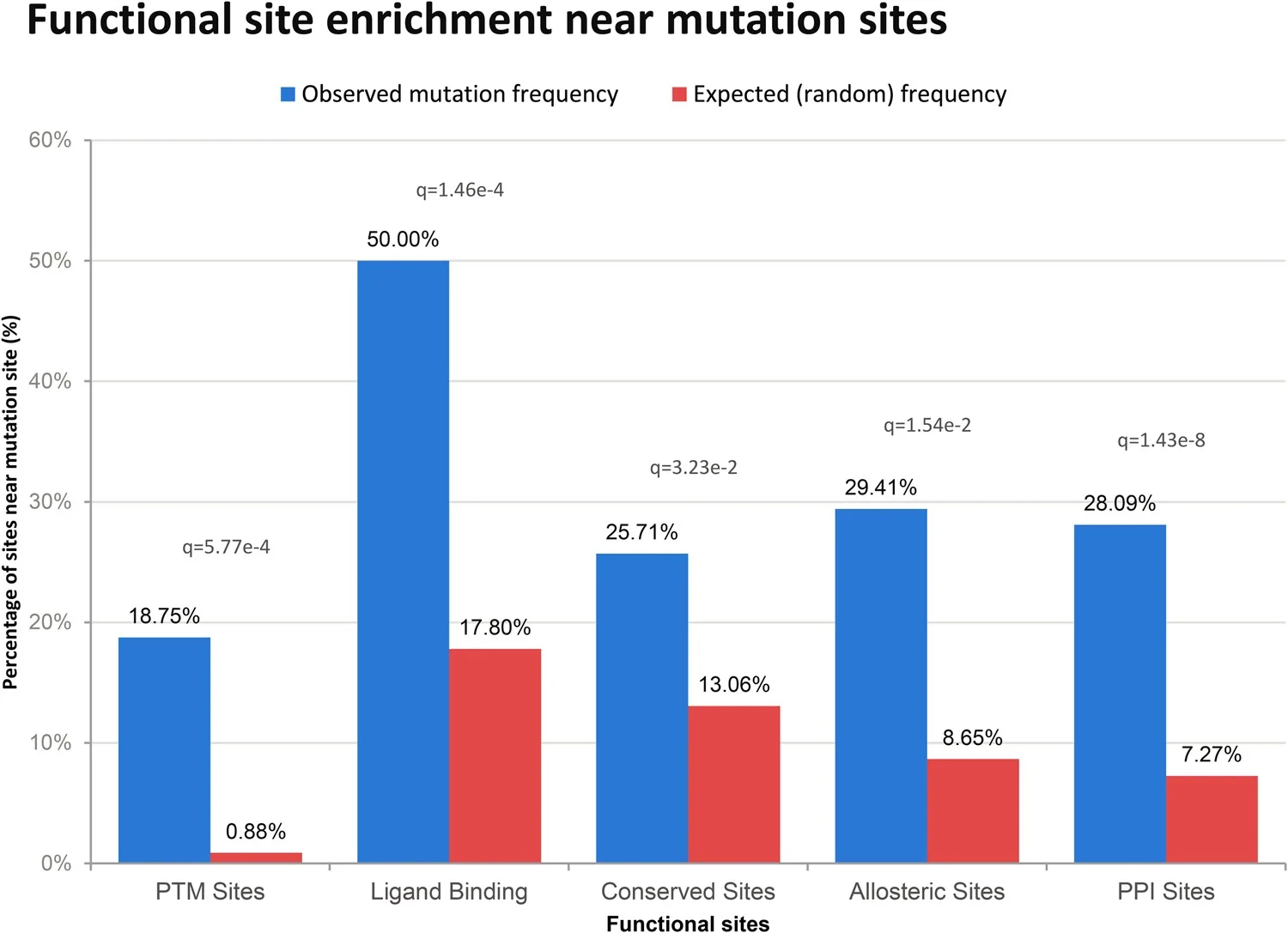
Detailed enrichment statistics, including observed and expected mutation frequencies, fold enrichment values, and FDR-adjusted *q*-values.

Protein–protein interface sites showed the strongest statistical support, followed by ligand-binding, PTM-associated, allosteric, and conserved regions, highlighting a significant accumulation of mutations within functional protein features. To our knowledge, this is the first comprehensive application of domain-level GWAS mapping to 3D structure prediction in plants, revealing that stress-responsive mutations show non-random enrichment in protein at functional protein regions, a pattern previously documented only in human disease mutations that caused structural alterations. A study by Sen *et al.* found that 60% of disease-associated human mutations occur near functional sites. Therefore, to validate our findings, we compared the distribution of functional site enrichment in rice stress-associated mutations with documented patterns in human disease-associated mutations (Sen *et al.* 2022). Our data showed similar preferential localization to protein–protein interface sites (28% versus 22%) suggesting that stress-responsive mutations may follow evolutionary constraints to those documented in human genetics. Notably, rice stress mutations showed higher enrichment at ligand-binding sites (50% versus 9%), suggesting potential functional adaptation through regulatory site modification under stress conditions.

To further strengthen our study, we performed a brief sensitivity analysis exploring alternative distance thresholds (3 Å, 4 Å, 6 Å, 7 Å, and 8 Å) to classify whether a certain mutation is close to a functional site. Our analysis revealed that the classification of 39 mutations near functional sites remained relatively stable between 4 and 5 Å thresholds (25 mutations in protein–protein interface sites), while broader thresholds (≥6 Å) substantially increased the count (48–53 mutations), risking over-inclusion of spatially distant residues (see Fig. 3). We infer that the 5 Å threshold thus represents a well-established compromise between capturing functionally relevant spatial interactions and avoiding arbitrary assignment of distant residues. It is also a commonly  used threshold used by the structural biology community, as this distance encompasses typical hydrogen bonding interactions (2.5–3.5 Å) and van der Waals contacts (∼4 Å), representing biologically relevant spatial relationships (Bordin *et al.* 2023, Waman *et al.* 2024).

**Figure 3:**
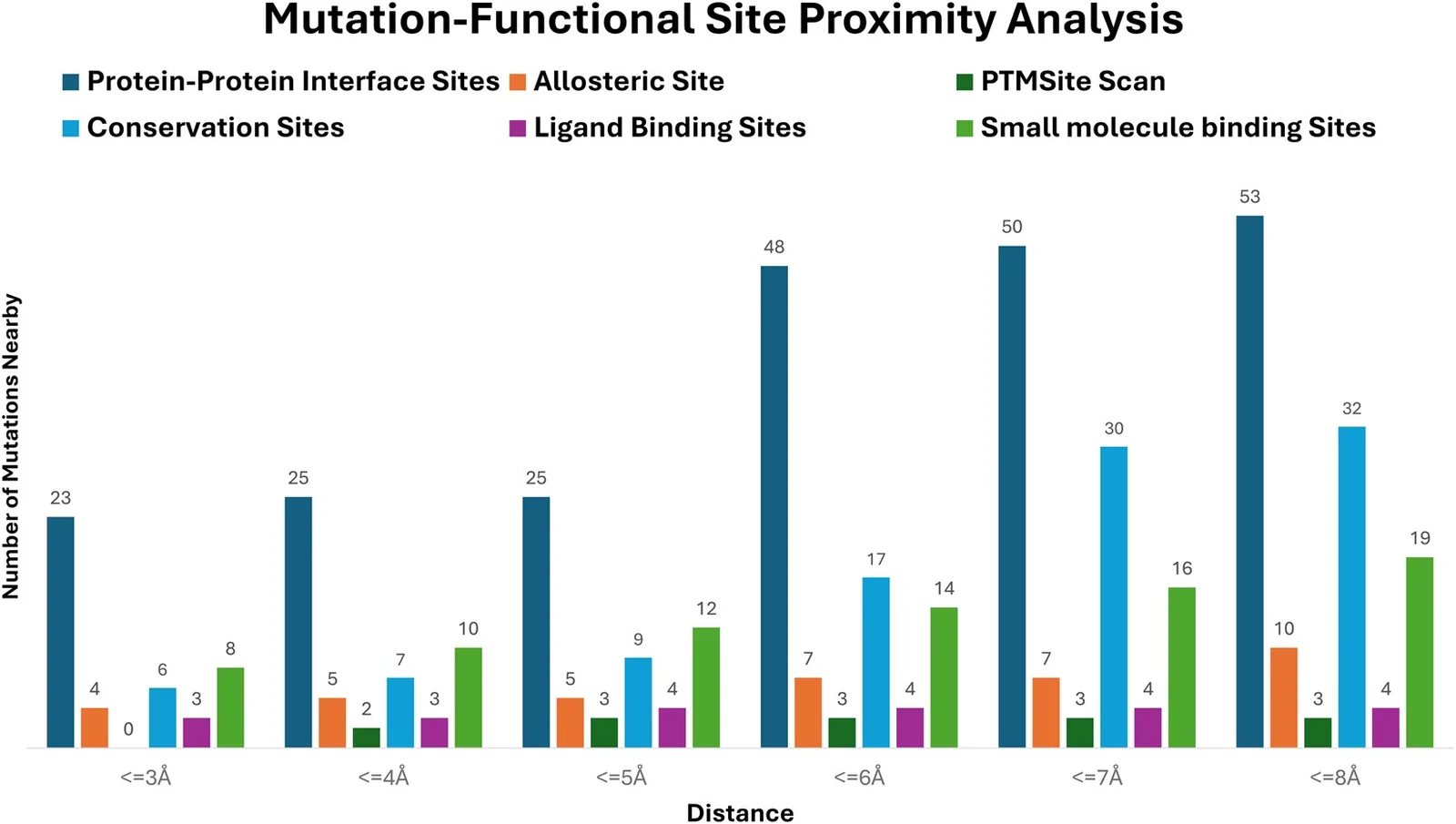
Mutation-functional site proximity analysis.

### 3.5 Selected examples of mutations likely to have an impact on phenotypic traits

We selected some examples (see Fig. 4) for more detailed analysis. Table 3 summarizes information about the chosen examples and their closest functional sites.

**Figure 4:**
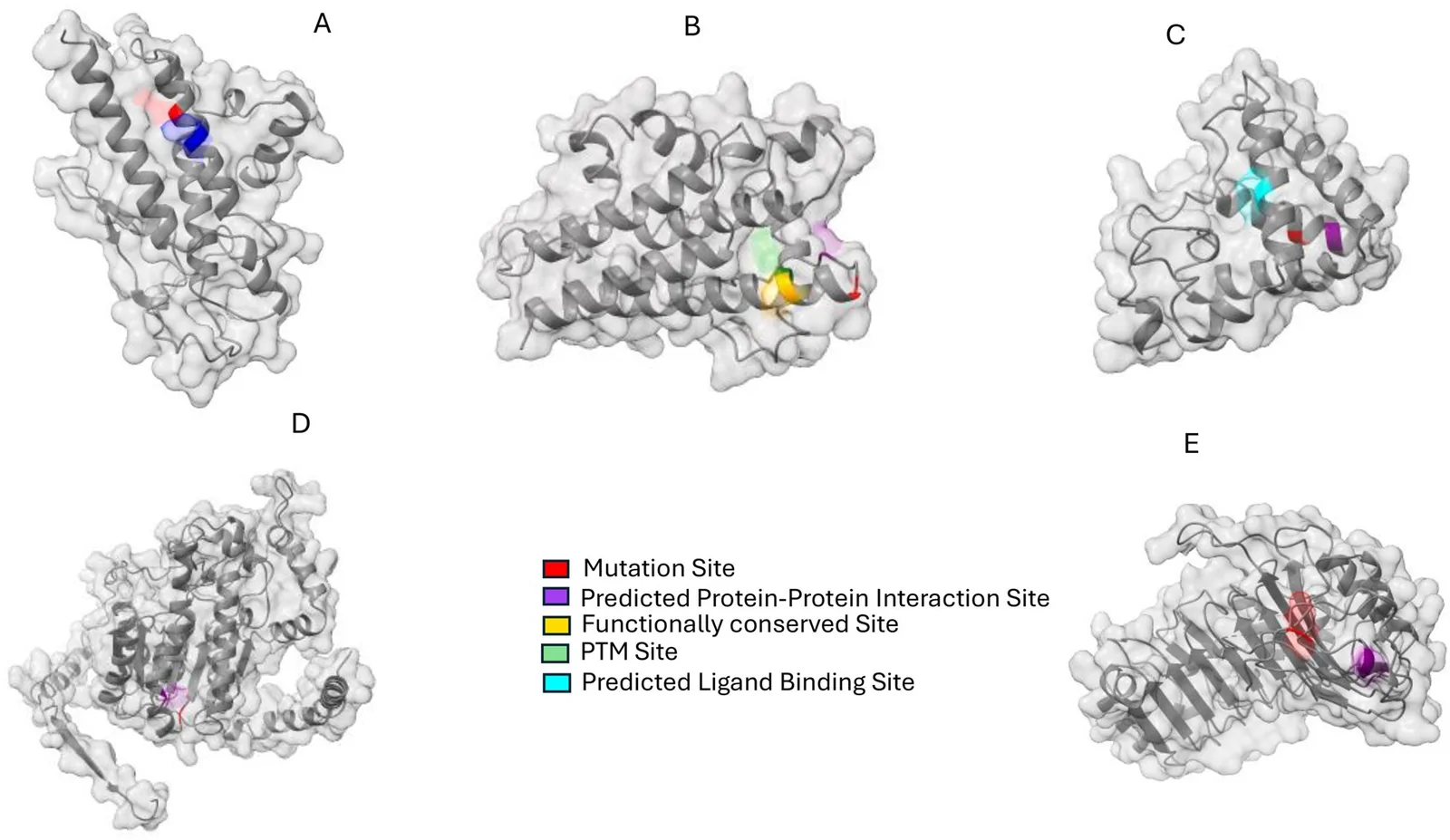
Example domains with mutations located close to predicted functional sites (A) A0A0P0Y5G5_8_175, (B) A0A0P0Y5N5_500_684, (C) Q5Z8H5_47_172_318_329, (D) Q6K785_39_349, and (E) Q2R078_99_147_165_422. Predicted functional site clusters located near the mutation are colour-coded as shown.

**Table 3:** Examples of mutations and their closest functional sites.

#	UniProt ID	Length	pLDDT	Mutation	GS	Stress
**1**	A0A0P0Y5G5	168	88	R130C	180	Biotic
Closest site: allosteric site 133 (3.1 Å)						
**2**	A0A0P0Y5N5	185	84	H527Y	83	Biotic
Closest sites: PTM 521 (5 Å); conserved site 522 (2.7 Å); ligand-binding and protein–protein interaction site 530 (2.9 Å)						
**3**	Q5Z8H5	138	95.8	A116T	58	Biotic
Closest sites: ligand-binding site 112 (3.1 Å); protein–protein interaction site 141 (3.3 Å)						
**4**	Q6K785	311	91.6	G191A	60	Abiotic
Closest site: protein–protein interaction and ligand-binding site 192 (1.3 Å)						
**5**	Q2R078	307	88	Q104K	53	Biotic
Closest site: protein–protein interaction site 107 (1.3 Å)						

#### 3.5.1 Mutations in transferases associated with bacterial blight disease

Os11g0690733 protein; UniProt ID A0A0P0Y5G5 (Fig. 4A) is annotated as a transferase in CATH-Gene3D (CATH superfamily ID: 1.10.510.10) and has been linked to bacterial blight disease resistance (Shu *et al.* 2021). Within this domain, the R130C mutation introduces a radical change in both side chain bulkiness and charge, which is likely to alter protein structure and function. Importantly, this mutation lies in proximity (3.08 Å from residue 133) to a PASSer-predicted allosteric site, suggesting potential functional consequences through altered allosteric regulation. The biological relevance of this finding is underscored by the findings of Dorion *et al.* (2021), who emphasized the role of transferases in redox balance and defence signalling/stress responses. The Os11g0694200 protein, UniProt ID A0A0P0Y5N5 (Fig. 4B) is another transferase, linked to bacterial blight disease resistance in rice, possessing an H527Y mutation (MutPred2 score 0.73, suggesting high impact). This mutation is close to a predicted PTM site (5 Å from residue 521), a functionally conserved site (2.76 Å from residue 522), and a small-molecule-binding and protein–protein interaction site (2.91 Å from residue 530). Protein–protein interaction analysis (see Figs 5A and 6A) showed that this transferase is predicted to interact with a serine/threonine protein kinase (Q8RUE8), mirroring defence pathway signalling (STRING score 0.5). This predicted interaction is supported by several computational indicators: the ipTM score is 0.78, while the pTM score is 0.51. The average pLDDT value is 70.61, indicating a reasonably confident structure prediction. The interaction shows a PRODIGY binding affinity score of −8.4 kcal/mol, suggesting favourable binding. Additionally, the PIZSA score is 1.6, and the mCSM-PPI2 ΔΔ*G* affinity value is 0.948 kcal/mol, further supporting the likelihood of a significant protein–protein interaction and increased affinity upon mutation.

**Figure 5:**
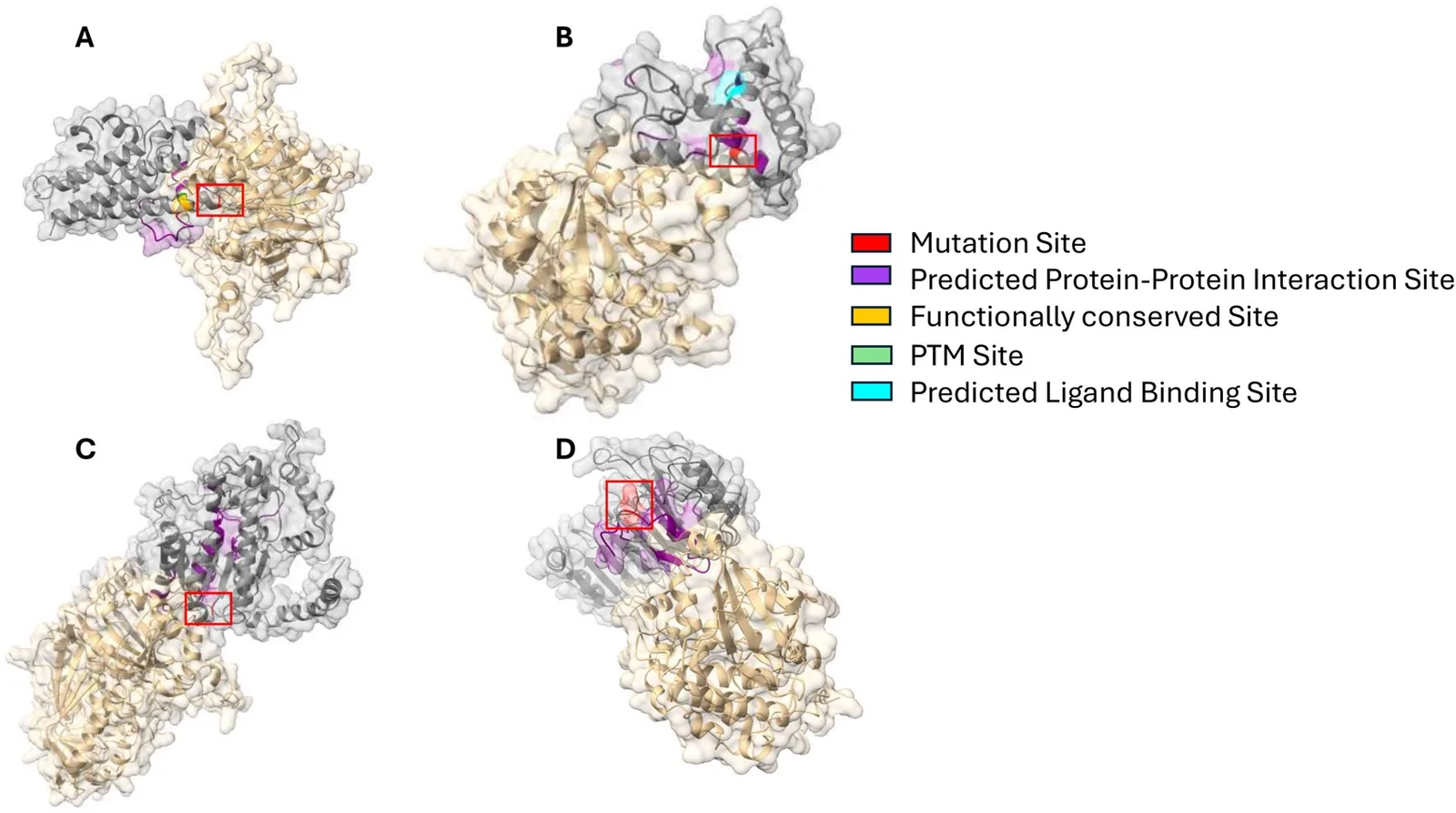
Interaction analyses of selected stress-associated mutations. (A) A0A0P0Y5N5_500_684 with interactor Q8RUE8; (B) af_Q5Z8H5_47_172_318_329 with interactor Q7XWU3; (C) Q6K785_39_349 with interactor Q2QRX6; and (D) Q2R078_99_147_165_422 with interactor Q10EP9. Purple patches depict the entire (top-scoring) predicted protein–protein interaction surface surrounding the mutation, as predicted by Cons-PPISP (Leis *et al*. 2010).

**Figure 6:**
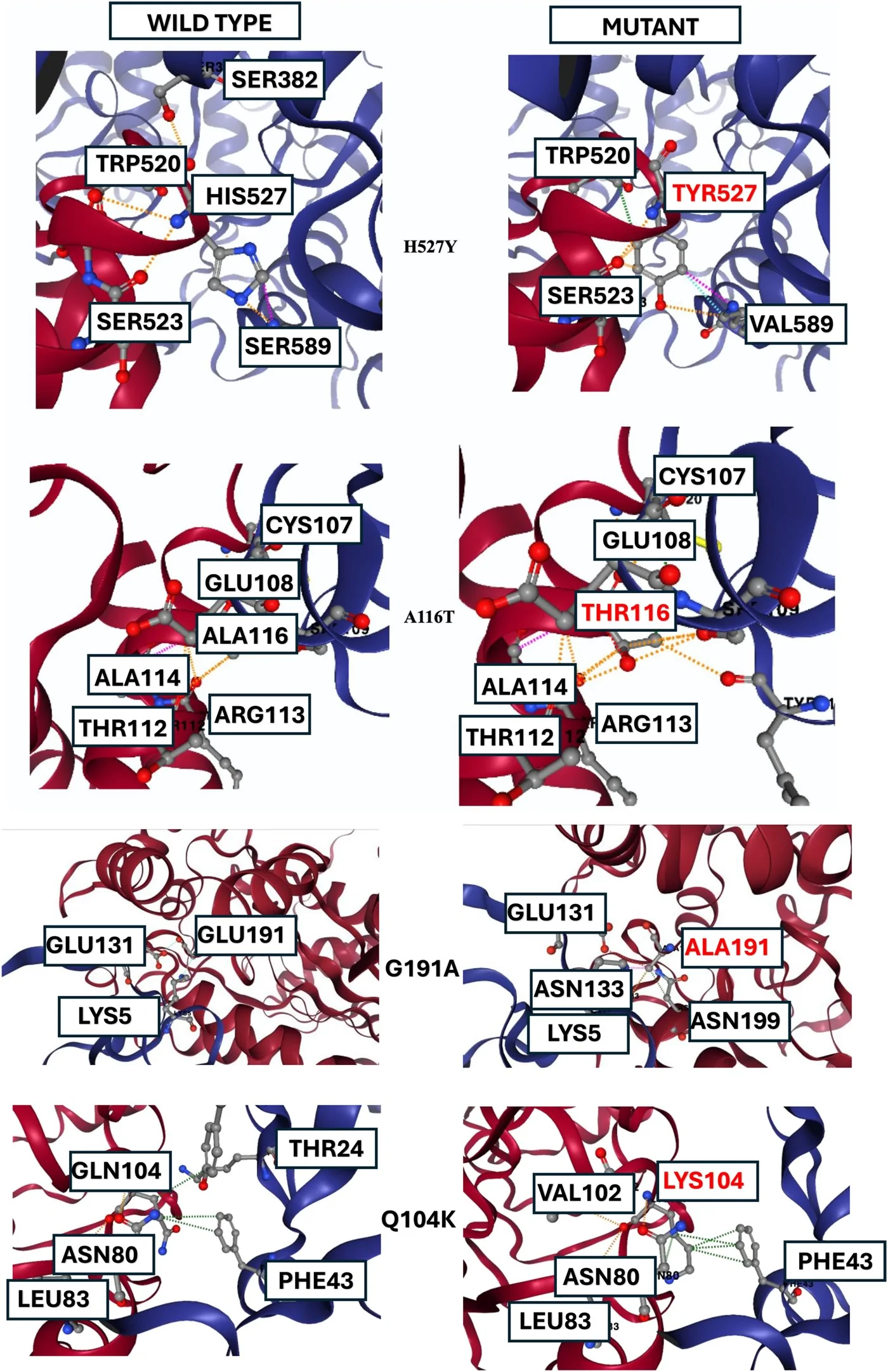
Detailed interaction analyses of selected stress-related mutations. Both wild and mutant types are shown with their respective interactors: (A) A0A0P0Y5N5_500_684 (mutation H527Y) with interactor Q8RUE8, (B) af_Q5Z8H5_47_172_318_329 (mutation A116T) with interactor Q7XWU3, (C) Q6K785_39_349 (mutated G191A) with interactor Q2QRX6, (D) Q2R078_99_147_165_422 (mutation Q104R) with interactor Q10EP9. (Red: query protein domain, Blue: predicted partner, Red dotted lines: hydrogen bonds, Orange dotted lines: polar interactions, Sky Blue for carbonyl bonds, hydrophobic bonds in green, pink dotted lines for clashes). The images were generated by mCSM-PPI2 (Pettersen *et al*. 2004, Rodrigues *et al*. 2019, Zhou *et al*. 2023, Zhou *et al*. 2024).

#### 3.5.2 Mutation in a peroxidase associated with bacterial blight disease

Another interesting example is a domain from a peroxidase protein, UniProt ID Q5Z8H5 (Fig. 4C), classified in the CATH peroxidase superfamily (1.10.520.10). Our analysis shows that an A116T mutation in this domain is associated with sheath blight disease resistance. This mutation involves a change from a hydrophobic to a hydrophilic residue and is close to a predicted ligand-binding site (3 Å from residue 112) and a protein–protein interface residue (3 Å from residue 141). Sahu *et al.* showed the involvement of this site in binding ligands like H_2_O_2_, in response to pathogen-induced stress and oxidative stress, suggesting a potential role in defence against the pathogen *Pseudomonas syringae* that causes bacterial blight disease (Sahu *et al.* 2022). The predicted interaction between the peroxidase domain and its predicted interactor, CAD6 (Cinnamyl Alcohol Dehydrogenase; UniProt ID Q7XWU3) has a STRING score of 0.84 (highly probable interaction) is supported by various metrics reporting the quality of the predicted complex (pLDDT 83.40, PIZSA *Z*-score: 1.73, ipTM 0.74 and pTM 0.55). Furthermore, PRODIGY predicted a negative free energy change (Δ*G*) of −11.4 kcal/mol, indicating energetically favourable binding. Using the complex structure, we calculated the predicted affinity change following mutation (A116T) using mCSM-PPI2 and obtained a predicted ΔΔ*G* affinity of 1.233 kcal/mol. An additional hydrogen bond is formed in the mutated variant (see Figs 5B and 6B). In other studies, CADs have also been found to be the strongest interactors with peroxidases, under stress conditions (Martínez-Cortés *et al.* 2021).

#### 3.5.3 Mutation in an NAD(P)-binding Rossmann-like domain associated with changes in seedling numbers following salt stress

Another mutation studied was that of a G191A mutation observed in an NAD(P)-binding Rossmann-like domain (UniProt ID Q6K785, Os02g0511400 protein) (Fig. 4D), which is associated with changes in seedling numbers following salt stress. This mutation is close to a predicted protein–protein interaction and a ligand-binding site (∼1 Å from residue 192). This domain is predicted to interact favourably with a putative bifunctional dihydrofolate reductase (DHFR, UniProt ID Q2QRX6), supported by a high STRING score (0.87). Analysis shows a predicted ipTM score of 0.73 and a pTM score of 0.55, indicating potential binding affinity. The average pLDDT score is 74.4, suggesting a good-quality model. Binding affinity and interface evaluation reveal a PRODIGY score of −14 kcal/mol, a PIZSA score of 1.24, and an mCSM-PPI2 ΔΔ*G* affinity of 0.847 kcal/mol in the variant complex, supporting a favourable interaction between the two proteins. In the mutated form, additional hydrophobic contacts form between the two interactors. See Figs 5C and 6C for the additional hydrophobic contacts in the mutant version.

#### 3.5.4 Ribonuclease inhibitor domain associated with yellow mottle virus resistance

We also analysed a mutation in a domain from a Leucine Rich Repeat protein, UniProt ID Q2R078 that belongs to the CATH ribonuclease inhibitor superfamily (3.80.10.10) and is also annotated by PANTHER as a leucine-rich repeat domain associated with  biotic stress in plants (Dickson *et al.* 2005) (Fig. 4E). The GWAS studies associated this protein with yellow mottle virus resistance. Leucine-rich repeat domains are well known to be involved in stress modulation in plants (Tyagi and Upadhyay 2023). The mutation Q104K is close (∼1 Å) to residue 107, which is a predicted protein–protein interaction site. It interacts with a serine/threonine kinase (UniProt ID Q10EP9) (STRING score of 0.515). The AlphaFold 3 predicted complex has scores of 0.53 for pTM, 0.61 for ipTM and an average pLDDT of 76.28. PRODIGY analysis reported a negative free energy change (Δ*G*) of −12.1 kcal/mol, indicating energetically favourable binding. The PIZSA score for the interaction is 1.62. All these values indicate a high-quality predicted protein complex. mCSM-PPI2 predicted an affinity change on mutation (Q104K) of ΔΔ*G* affinity of 1.216 kcal/mol and reported that the mutated residue participates in additional hydrogen bond formation (see Figs 5D and 6D). This interaction further supports the LRR protein family’s central role in plant stress signalling and defence adaptation (Soltabayeva *et al.* 2022).

The results are summarized in Fig. 7.

**Figure 7:**
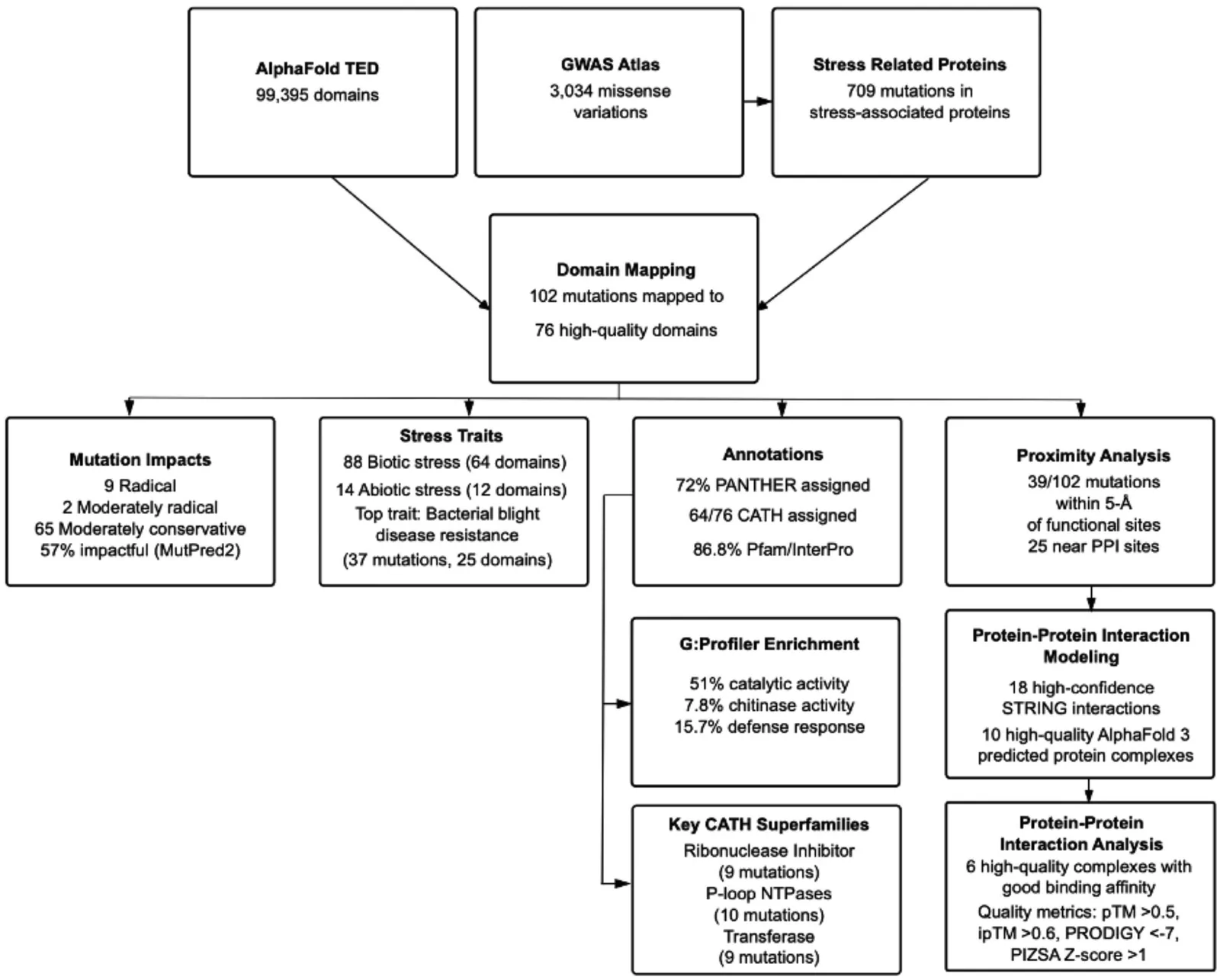
Summary of analyses of the impact of missense variants in plant proteins and their role in moderating stress.

For the 63 mutations (out of 102) not lying close to functional sites, FoldX 5.1 was used to evaluate whether the mutations were stabilizing or destabilizing the protein structure (Potapov *et al.* 2009). Forty-six percent (29 of 63) of these mutations were destabilizing (ΔΔ*G* > 0.5 kcal/mol), indicating a likely impact on protein stability. Among the remaining 54% (34 of 63) non-destabilizing variants, we note that a subset may represent highly stabilizing mutations (ΔΔ*G* < −0.5 kcal/mol), which could functionally matter through mechanisms such as enhanced expression, improved folding kinetics, or stabilization of catalytically competent conformations. Random model analysis of FoldX predicted 53.7% of the randomly generated mutations to be destabilizing (ΔΔ*G* > 0.5 kcal/mol), compared with 46% of the observed stress-associated mutations (see [AQ]S9b). This lower proportion of destabilizing mutations is consistent with the possibility that stress-associated mutations may be less likely to have strongly destabilizing effects than expected by chance. This pattern is consistent with evolutionary constraints favouring mutations that maintain sufficient protein stability and function. MutPred2 identified approximately one-third of these non-destabilizing variants (nine of 34; 26.5%) as potentially impactful. The remaining 24 (including a radical and six moderately radical) variants showed no predicted effects, which may reflect the limited sensitivity of current computational tools or inaccuracies in GWAS associations. It should be noted that our structure-based approach does not capture all mechanisms of protein function, including potential regulatory/allosteric effects or transient interactions that may influence how mutations affect protein stability.

Moreover, for those 63 variants without known function annotations, we identified homologous protein structures to determine whether the mutated residues were located near bound DNA/RNA molecules or at conserved water-binding sites, both of which may indicate functional importance.

A total of 38 of the 63 variants (∼60%) were successfully analysed; the remaining 25 (∼40%) had no homolog available at this threshold. Of the 38 testable variants, three were located near a conserved water site; UniProt ID A0A0P0X1N3; site L187G (conservation score = 0.80), UniProt ID Q0D921; site M242T (conservation score = 0.857), UniProt ID Q0IQX8; site R227W (conservation score = 0.70), but none were located near a predicted DNA/RNA-binding residue.

We therefore suggest that most of these missense mutations may exert their effects through mechanisms not captured by proximity to classical functional sites, such as indirect structural effects or regulatory mechanisms, or these results could probably be a result of GWAS errors. See Table 10, available as supplementary data at *Bioinformatics Advances* online for a summary of the impact assessment for all those mutations that do not have a predicted functional site within 5 Å.

This study employs a comprehensive computational framework to analyse the structural and functional consequences of protein variation in plants, with several considerations relevant to interpretation. Experimentally resolved protein structures are unavailable for many plant proteins; accordingly, the analysis uses TED domain predictions derived from high-confidence AlphaFold models (pLDDT > 70) to obtain domain-level structural information. These models are robust at the domain scale but do not explicitly represent conformational dynamics, cofactor-bound states, or context-dependent PTMs that may influence protein function *in vivo*. In addition, many plant proteins remain incompletely characterized with respect to molecular context, including oligomeric state, subcellular localization, and dominant splice isoforms. As a result, several interpretations rely on homology-driven inference, a common and necessary approach in large-scale plant protein analyses. Despite these constraints, TED enables systematic cross-species comparisons and the identification of conserved structural features in the absence of experimentally determined structures.

Protein–protein interaction predictions generated using AlphaFold3 represent computational models supported by multiple quality metrics (pTM, ipTM, PRODIGY, and PIZSA scores) and provide hypotheses regarding interaction interfaces and binding modes, which require experimental validation using approaches such as co-immunoprecipitation, yeast two-hybrid assays, or isothermal titration calorimetry to confirm binding affinities and assess mutation-induced effects. In parallel, mutation effect prediction tools including MutPred2, FoldX, and mCSM-PPI2 are well-established algorithms trained predominantly on human and model organism datasets and are useful for variant prioritization, although their performance has not yet been systematically benchmarked for plant proteins. Functional site predictions derived from complementary methods (PASSer, P2Rank, AlphaFill, and Cons-PPISP) provide convergent computational evidence for residues of potential functional relevance.

This study presents a systematic computational analysis that generates prioritized, testable hypotheses regarding the effects of mutations on protein function. These hypotheses now require experimental validation through targeted mutagenesis studies, biochemical assays, and phenotypic characterization in rice cultivars to transition from predictive insights to confirmed functional mechanisms. As plant structural resources continue to expand, these predictions offer a strong foundation for future experimental validation and refinement, demonstrating the value of computational proteomics for accelerating crop improvement research in an era when experimental validation of thousands of variants remains resource-intensive, while also providing a robust framework for identifying promising candidates for experimental follow-up.

While our computational framework leverages multiple predictive tools to annotate putative functional sites, we acknowledge that these tools were primarily trained on experimentally resolved non-plant protein structures, and their error rates when applied to plant systems remain incompletely characterized. Functional site annotations were derived from machine-learning predictors applied to AlphaFold2-generated models and should be interpreted as predictions rather than as established experimental facts. Notably, ligand-binding site prediction using P2Rank achieves approximately 68.6–72.0% accuracy across two datasets, indicating that 27–32% of true binding sites may be missed, or false positives may be included. For allosteric site prediction, PASSer employs ensemble learning with 0.726 precision, 0.847 recall, and an *F*1 score of 0.782, ranking actual allosteric sites in the top 1 and top 3 positions 60.7% and 84.9% of the time, respectively, indicating that approximately 15% of true allosteric sites may not be identified. Cons-PPISP, which integrates multiple neural network models, employs a probability threshold of >0.5 to balance sensitivity and specificity and achieves 69% accuracy with 47% coverage on experimentally characterized protein complexes (Jordan *et al.* 2012, Krivák and Hoksza 2018).

Notably, a major unaddressed challenge is how local model confidence from AlphaFold2 (pLDDT scores) propagates through these predictions: functional sites inherit uncertainty from the underlying protein models, and predicted sites located in low-confidence regions (pLDDT < 70) should be treated with heightened caution. We still recommend validating high-priority predicted sites through orthogonal approaches (mutagenesis, biochemical assay, or structural refinement) before functional assignment, and interpreting results as ranked hypotheses for experimental follow-up rather than definitive functional annotations.

## 4 Conclusion

This study employed a structural bioinformatics pipeline, exploiting accurately predicted protein structures from the AlphaFold Protein Structure Database, to analyse the impact of missense variants in rice proteins and their role in moderating stress. A total of 77 787 high-quality domains were considered, with 102 mutations associated with stress resistance were mapped. Key findings indicate that 14 mutations in 12 domains are linked to abiotic stress tolerance, while 88 mutations in 64 domains are associated with biotic stress, with 37 mutations in 25 domains explicitly related to bacterial blight resistance. Notably,  39/102 mutations are located close to predicted protein functional sites, suggesting their potential role in the stress response. The study also demonstrated how these mutations could impact the binding affinity to partner proteins. This was done by modelling the interactions between 18 protein pairs using AlphaFold 3, with six mutations predicted to enhance interaction affinity with the protein partners.

However, whilst providing insights on mutations that may influence stress response, the research underscores the critical need for further experimental validation to fully understand the connection between plant protein variations and stress response. It also highlights the paucity of plant data compared with data from human and other model organisms, which poses significant challenges. In addition, it demonstrates the need for specialized mutation-effect prediction tools trained on plant-specific data to overcome current limitations. Addressing these challenges will require collaborative efforts and advanced technologies aimed at generating, characterizing, and analysing reliable plant data.

## Supplementary Material

vbag275_Supplementary_Data

## Data Availability

The data underlying this article are available in the Supplementary Materials.
