# Supplementary material for: Employing AlphaFold and TED to explore the structural impacts of stress-associated mutations in rice
Source: Bioinform Adv. 2026 Sep 22;6(1):vbag275. doi: 10.1093/bioadv/vbag275 (PMC13652148; doi:10.1093/bioadv/vbag275)
Supplement: Supplementary file 1 [file vbag275_supplementary_data.docx]

**Supplementary file**

**S1: Rice structural domains along with the impactful mutations and GWAS traits**

| **Domain** | **Traits** | **Substitution** | **length of domain** | **Q score** | **Grantham** | **MutPred2 score** |
| --- | --- | --- | --- | --- | --- | --- |
| A0A0N7KIR5_4_111 | Brown Plant Hopper Resistance | A71T | 108 | 87.96 | 58 | 0.159 |
| A0A0N7KU10_166_320 | rice Bacterial Blight Disease Resistance | S263L | 147 | 84.1 | 180 | 0.044 |
| A0A0P0VJK1_180_396 | seedling numbers changed due to salt stress | L275P | 217 | 95.47 | 102 | 0.164 |
| A0A0P0VJK1_180_396 | seedling numbers changed due to salt stress | L210R | 217 | 95.47 | 98 | 0.106 |
| A0A0P0VR33_241_308_327_422 | cold tolerance | A366T | 164 | 91.06 | 58 | 0.206 |
| A0A0P0W110_218_425 | rice yellow mottle virus resistance | T243I | 208 | 76.96 | 89 | 0.331 |
| A0A0P0W767_67_224 | Brown Plant Hopper Resistance | H81D | 158 | 92.96 | 81 | 0.238 |
| A0A0P0WSE1_45_199 | Brown Plant Hopper Resistance | P121R | 155 | 82.42 | 145 | 0.653 |
| A0A0P0WSE1_45_199 | Brown Plant Hopper Resistance | N67Y | 155 | 82.42 | 143 | 0.605 |
| A0A0P0WSE1_45_199 | Brown Plant Hopper Resistance | L98R | 155 | 82.42 | 126 | 0.561 |
| A0A0P0WSE1_45_199 | Brown Plant Hopper Resistance | T69I | 155 | 82.42 | 103 | 0.558 |
| A0A0P0WSE1_45_199 | Brown Plant Hopper Resistance | A56D | 155 | 82.42 | 102 | 0.472 |
| A0A0P0WSE1_45_199 | Brown Plant Hopper Resistance | E54K | 155 | 82.42 | 94 | 0.355 |
| A0A0P0WSE1_45_199 | Brown Plant Hopper Resistance | N125K | 155 | 82.42 | 89 | 0.339 |
| A0A0P0WSE1_45_199 | Brown Plant Hopper Resistance | Q122K | 155 | 82.42 | 64 | 0.193 |
| A0A0P0WSE1_45_199 | Brown Plant Hopper Resistance | S106L | 155 | 82.42 | 56 | 0.136 |
| A0A0P0WSE1_45_199 | Brown Plant Hopper Resistance | A112V | 155 | 82.42 | 53 | 0.104 |
| A0A0P0X1N3_13_195_202_226 | Sheath Blight Disease Resistance | L187G | 208 | 76.83 | 138 | 0.909 |
| A0A0P0X1S7_7_245 | Sheath Blight Disease Resistance | N187G | 239 | 76.91 | 81 | 0.624 |
| A0A0P0X1S7_7_245 | Sheath Blight Disease Resistance | T247M | 239 | 76.91 | 80 | 0.461 |
| A0A0P0X776_25_126 | Anaerobic response index | E42G | 102 | 94.36 | 127 | 0.33 |
| A0A0P0XHS6_50_353 | Sheath Blight Disease Resistance | T226I | 304 | 89.81 | 89 | 0.761 |
| A0A0P0XSV6_275_288_423_521_531_662 | Sheath Blight Disease Resistance | R542C | 245 | 76.07 | 180 | 0.211 |
| A0A0P0Y553_175_243 | rice Bacterial Blight Disease Resistance | S202L | 69 | 86.62 | 145 | 0.272 |
| A0A0P0Y5B0_9_159_324_373 | rice Bacterial Blight Disease Resistance | W120L | 186 | 76.43 | 56 | 0.853 |
| A0A0P0Y5G5_8_175 | rice Bacterial Blight Disease Resistance | E116K | 168 | 81.73 | 180 | 0.702 |
| A0A0P0Y5G5_8_175 | rice Bacterial Blight Disease Resistance | R130C | 168 | 81.73 | 56 | 0.298 |
| A0A0P0Y5N1_5_124 | rice Bacterial Blight Disease Resistance | K29E | 120 | 91.82 | 99 | 0.607 |
| A0A0P0Y5N1_5_124 | rice Bacterial Blight Disease Resistance | H85L | 120 | 91.82 | 98 | 0.29 |
| A0A0P0Y5N1_5_124 | rice Bacterial Blight Disease Resistance | G119E | 120 | 91.82 | 56 | 0.168 |
| A0A0P0Y5N5_500_684 | rice Bacterial Blight Disease Resistance | H527Y | 185 | 92.36 | 83 | 0.734 |
| A0A0P0Y5Q7_33_298 | rice Bacterial Blight Disease Resistance | A179T | 266 | 97.62 | 58 | 0.652 |
| A0A0P0Y5Q8_99_222 | rice Bacterial Blight Disease Resistance | T220A | 124 | 81.61 | 58 | 0.264 |
| A0A0P0Y5U5_89_103_112_247 | rice Bacterial Blight Disease Resistance | S234R | 151 | 90.06 | 143 | 0.495 |
| A0A0P0Y5U5_89_103_112_247 | rice Bacterial Blight Disease Resistance | N220Y | 151 | 90.06 | 110 | 0.343 |
| A0A0P0Y5U5_89_103_112_247 | rice Bacterial Blight Disease Resistance | A232S | 151 | 90.06 | 99 | 0.26 |
| A0A5S6R7C8_49_268_285_318_337_379_392_409 | Brown Plant Hopper Resistance | K138N | 315 | 91.5 | 94 | 0.282 |
| A3BFX5_1_144 | Sheath Blight Disease Resistance | H25N | 144 | 87.49 | 68 | 0.167 |
| B9FA56_221_429 | Anaerobic response index | A304T | 209 | 91.67 | 58 | 0.06 |
| B9FR93_19_115_131_191 | Brown Plant Hopper Resistance | C164S | 158 | 76.62 | 155 | 0.391 |
| B9FR93_19_115_131_191 | Brown Plant Hopper Resistance | F184S | 158 | 76.62 | 112 | 0.148 |
| C7IZM0_51_156_219_258 | Sheath Blight Disease Resistance | D236R | 146 | 97.33 | 96 | 0.827 |
| C7J8J8_12_128 | rice Bacterial Blight Disease Resistance | L83R | 117 | 97.62 | 102 | 0.686 |
| C7J8J8_12_128 | rice Bacterial Blight Disease Resistance | N41T | 117 | 97.62 | 65 | 0.235 |
| O49826_30_284 | rice Bacterial Blight Disease Resistance | K163N | 255 | 96.93 | 94 | 0.747 |
| Q0D921_167_606 | Sheath Blight Disease Resistance | M242T | 440 | 80.41 | 81 | 0.745 |
| Q0D921_167_606 | Sheath Blight Disease Resistance | D187G | 440 | 80.41 | 94 | 0.7 |
| Q0DIJ7_259_434 | Seedling Survival Days Associated to Salinity Tolerance | M287T | 176 | 87.3 | 60 | 0.475 |
| Q0DIJ7_259_434 | Seedling Survival Days Associated to Salinity Tolerance | G298A | 176 | 87.3 | 81 | 0.149 |
| Q0DQB4_57_217 | rice yellow mottle virus resistance | W58T | 283 | 87.59 | 128 | 0.896 |
| Q0IMT5_12_102_160_234_341_418 | Total Lesion Length induced by Sheath Blight | K210N | 244 | 87.56 | 94 | 0.476 |
| Q0IP79_169_317 | Sheath Blight Disease Resistance | D183V | 149 | 88.37 | 152 | 0.424 |
| Q0IQX8_32_277 | rice Bacterial Blight Disease Resistance | R227W | 246 | 89.86 | 101 | 0.478 |
| Q0IR17_30_39_53_154 | rice Bacterial Blight Disease Resistance | L129G | 155 | 73.93 | 96 | 0.901 |
| Q0IR17_30_39_53_154 | rice Bacterial Blight Disease Resistance | R57A | 112 | 73.93 | 134 | 0.804 |
| Q0IR17_30_39_53_154 | rice Bacterial Blight Disease Resistance | D82K | 112 | 73.93 | 101 | 0.724 |
| Q0IR17_30_39_53_154 | rice Bacterial Blight Disease Resistance | I103T | 112 | 73.93 | 138 | 0.538 |
| Q0IR17_30_39_53_154 | rice Bacterial Blight Disease Resistance | I128E | 112 | 73.93 | 89 | 0.48 |
| Q0IR17_30_39_53_154 | rice Bacterial Blight Disease Resistance | F73A | 112 | 73.93 | 112 | 0.27 |
| Q0IR17_45_199 | rice Bacterial Blight Disease Resistance | L116A | 112 | 73.93 | 113 | 0.752 |
| Q0IR63_201_321 | rice Bacterial Blight Disease Resistance | G311S | 121 | 77.82 | 56 | 0.884 |
| Q0IR75_175_322 | rice yellow mottle virus resistance | A201T | 148 | 94.25 | 58 | 0.206 |
| Q0IR99_2_148_160_321 | rice Bacterial Blight Disease Resistance | S59A | 309 | 98.28 | 99 | 0.135 |
| Q0IVA2_17_222 | rice yellow mottle virus resistance | S69P | 206 | 90.68 | 74 | 0.682 |
| Q0JEW8_64_395 | Brown Plant Hopper Resistance | A254G | 332 | 87.34 | 60 | 0.438 |
| Q0JG81_31_201 | Sheath Blight Disease Resistance | N52K | 171 | 98.76 | 94 | 0.212 |
| Q0JH25_516_793 | anaerobic response index | L793S | 278 | 90.19 | 145 | 0.822 |
| Q10MH8_189_424 | anaerobic response index | P217S | 236 | 96.05 | 74 | 0.836 |
| Q2QP28_13_60_163_233_342_417 | Total Lesion Length induced by Sheath Blight | K210N | 195 | 95.25 | 94 | 0.538 |
| Q2QR00_166_319 | rice Bacterial Blight Disease Resistance | T305S | 154 | 84.09 | 58 | 0.092 |
| Q2QV98_171_319 | Sheath Blight Disease Resistance | D183V | 149 | 84.51 | 152 | 0.424 |
| Q2QZH9_485_562 | seedling numbers changed due to salt stress | S501T | 78 | 79.18 | 58 | 0.178 |
| Q2QZP4_23_137 | rice Bacterial Blight Disease Resistance | R49C | 115 | 79.26 | 180 | 0.291 |
| Q2QZS0_174_325 | rice Bacterial Blight Disease Resistance | H321Y | 152 | 90.78 | 83 | 0.188 |
| Q2QZV8_176_320 | rice yellow mottle virus resistance | A201T | 145 | 94.8 | 58 | 0.206 |
| Q2R037_2_321 | rice Bacterial Blight Disease Resistance | S59A | 320 | 98.31 | 99 | 0.135 |
| Q2R077_59_355 | rice yellow mottle virus resistance | P85S | 297 | 92.83 | 74 | 0.579 |
| Q2R078_99_147_165_422 | rice yellow mottle virus resistance | Q104K | 307 | 85.56 | 53 | 0.115 |
| Q2RBQ7_46_226 | rice yellow mottle virus resistance | S69P | 181 | 81.06 | 74 | 0.682 |
| Q53NL2_33_279 | rice Bacterial Blight Disease Resistance | R227W | 247 | 96.39 | 101 | 0.478 |
| Q53NL3_32_285 | rice Bacterial Blight Disease Resistance | K163N | 254 | 96.56 | 94 | 0.747 |
| Q53NL9_35_299 | rice Bacterial Blight Disease Resistance | A179T | 265 | 97.22 | 58 | 0.652 |
| Q53QC7_513_710 | rice Bacterial Blight Disease Resistance | C591S | 198 | 96.84 | 143 | 0.631 |
| Q53QC7_513_710 | rice Bacterial Blight Disease Resistance | N686Y | 198 | 96.84 | 112 | 0.381 |
| Q5VQ96_13_96_116_201_426_452 | Brown Plant Hopper Resistance | A185T | 212 | 74.69 | 58 | 0.416 |
| Q5VQ96_505_716 | Brown Plant Hopper Resistance | K638T | 212 | 74.69 | 78 | 0.436 |
| Q5Z8H5_47_172_318_329 | Sheath Blight Disease Resistance | A116T | 115 | 86.64 | 58 | 0.718 |
| Q67VR7_27_366 | Seedling Survival Days Associated to Salinity Tolerance | R302C | 340 | 95.39 | 180 | 0.315 |
| Q69X07_347_464 | nitrogen sensitivity | G423R | 118 | 76.66 | 125 | 0.217 |
| Q6K785_39_349 | seedling numbers changed due to salt stress | G191A | 311 | 84.8 | 60 | 0.387 |
| Q6K973_161_186_195_274 | Total Lesion Length induced by Sheath Blight | N224H | 106 | 94.08 | 68 | 0.184 |
| Q6ZDZ0_482_491_500_559_585_693 | Sheath Blight Disease Resistance | R672W | 179 | 89.33 | 101 | 0.344 |
| Q75GX1_220_429 | anaerobic response index | A304T | 210 | 91.4 | 58 | 0.06 |
| Q75H81_191_287_354_390 | Sheath Blight Disease Resistance | G236R | 134 | 89.24 | 125 | 0.226 |
| Q7F8X6_11_249_456_482 | rice yellow mottle virus resistance | H21D | 266 | 97.82 | 81 | 0.612 |
| Q7FAZ3_21_151 | Brown Plant Hopper Resistance | R129C | 131 | 88.26 | 180 | 0.446 |
| Q7XSK4_63_393 | Brown Plant Hopper Resistance | A254G | 331 | 87.67 | 60 | 0.438 |
| Q84ZI6_430_646 | Blast disease resistance | A542T | 217 | 94.63 | 58 | 0.624 |
| Q8GS08_36_187 | Relative Salt Affected Root Surface Area | P61L | 152 | 87.95 | 98 | 0.419 |
| Q8H651_204_404 | Brown Plant Hopper Resistance | M206T | 201 | 94.93 | 81 | 0.177 |
| Q8W3E8_37_220 | nitrogen sensitivity | A123T | 184 | 86.59 | 58 | 0.19 |
| Q9FWT5_59_213 | rice Bacterial Blight Disease Resistance | D175G | 155 | 86.93 | 94 | 0.668 |

Note: Table also shows Trait analysis from GWAS Atlas, along with the filtered mutations involved in stress mechanisms

**S2a Comparative analysis of Grantham score distribution among different species**

| **# Mutations** | **Rice** | **Maize** | **Soybean** |
| --- | --- | --- | --- |
| Conservative | 70(40.6%) | 9 (40.9%) | 3(60%) |
| Moderately conservative | 65(38.2%) | 9(40.9%) | 2(40%) |
| Moderately radical | 28(16.2%) | 1(4.5%) | 0 |
| Radical | 9(5.2%) | 3(13.6%) | 0 |
| Total | 172 | 22 | 5 |

**S2b Comparison of observed and random rice missense mutations on the basis of Grantham score**

| **Grantham Score Category** | **Stress-associated mutations (%)** | **Random mutations (%)** |
| --- | --- | --- |
| Radical | 5.2 | 16.2 |
| Moderately radical | 16.3 | 29.0 |
| Moderately conservative | 37.8 | 36.9 |
| Conservative | 40.7 | 17.9 |

**S 3: PANTHER Annotation**

**S3: Panther annotation of mutation-containing protein domains**

| **Mapped IDs** | **Matched Domains** | **Orthologs** | **PANTHER Family/Subfamily** | **PANTHER Protein Class** |
| --- | --- | --- | --- | --- |
| Q75GX1 | Q75GX1_220_429 | Expressed protein | EXPRESSED PROTEIN (PTHR43019:SF29) | serine protease |
| Q6K973 | Q6K973_161_186_195_274 | Sucrose synthase 6 | SUCROSE SYNTHASE 6 (PTHR45839:SF16) | - |
| Q0JG81 | Q0JG81_31_201 | Os01g0937200 protein | OS01G0937200 PROTEIN (PTHR47965:SF63) | protease |
| A0A0P0VR33 | A0A0P0VR33_241_308_327_422 | 3-ketoacyl-CoA synthase | 3-KETOACYL-COA SYNTHASE (PTHR31561:SF7) | - |
| A0A0P0Y5N1 | A0A0P0Y5N1_5_124 | Os11g0690600 protein | - | - |
| Q2RBQ7 | Q2RBQ7_46_226 | Os11g0102700 protein | SENTRIN-SPECIFIC PROTEASE 8 (PTHR46468:SF1) | protease |
| Q6ZDZ0 | Q6ZDZ0_482_491_500_559_585_693 | Os07g0134600 protein | OS07G0134600 PROTEIN (PTHR45974:SF141) | transmembrane signal receptor |
| A0A0P0VJK1 | A0A0P0VJK1_180_396 | dihydrolipoyllysine-residue succinyltransferase | DIHYDROLIPOYLLYSINE-RESIDUE SUCCINYLTRANSFERASE COMPONENT OF 2-OXOGLUTARATE DEHYDROGENASE COMPLEX, MITOCHONDRIAL (PTHR43416:SF5) | transferase |
| A0A0N7KIR5 | A0A0N7KIR5_4_111 | Os04g0276400 protein | - | - |
| Q53QC7 | Q53QC7_513_710 | Os11g0694100 protein | OS11G0691500 PROTEIN (PTHR27005:SF162) | transmembrane signal receptor |
| Q69X07 | Q69X07_347_464 | Os06g0605900 protein | OS06G0605900 PROTEIN (PTHR13318:SF173) | - |
| Q9FWT5 | Q9FWT5_59_213 | Ripening-related protein 3 | RIPENING-RELATED PROTEIN 3 (PTHR33191:SF27) | - |
| Q2QP28 | Q2QP28_13_60_163_233_342_417 | Os12g0543600 protein | FAD DEPENDENT OXIDOREDUCTASE DOMAIN-CONTAINING PROTEIN (PTHR10961:SF7) | oxidase |
| A0A0P0Y5G5 | A0A0P0Y5G5_8_175 | Os11g0690733 protein | OS11G0691500 PROTEIN (PTHR27005:SF162) | transmembrane signal receptor |
| Q2QZP4 | Q2QZP4_23_137 | Expressed protein | - | - |
| Q75H81 | Q75H81_191_287_354_390 | Serpin-ZXA | GH10112P-RELATED (PTHR11461:SF211) | protease inhibitor |
| Q67VR7 | Q67VR7_27_366 | Leucoanthocyanidin dioxygenase 2 | 2-OXOGLUTARATE (2OG) AND FE(II)-DEPENDENT OXYGENASE SUPERFAMILY PROTEIN-RELATED (PTHR47990:SF167) | oxygenase |
| Q0JH25 | Q0JH25_516_793 | phosphoribosylformylglycinamidine synthase | PHOSPHORIBOSYLFORMYLGLYCINAMIDINE SYNTHASE (PTHR10099:SF1) | - |
| O49826 | O49826_30_284 | Chitinase | XYLANASE INHIBITOR PROTEIN 1 (PTHR45708:SF4) | - |
| Q8H651 | Q8H651_204_404 | Os06g0126250 protein | OS06G0126250 PROTEIN (PTHR27003:SF106) | - |
| Q2QV98 | Q2QV98_171_319 | NB-ARC domain containing protein, expressed | OS08G0205100 PROTEIN (PTHR23155:SF906) | antimicrobial response protein |
| A0A0P0XSV6 | A0A0P0XSV6_275_288_423_521_531_662 | Os10g0157000 protein | - | - |
| Q2QZS0 | Q2QZS0_174_325 | NB-ARC domain containing protein, expressed | OS11G0676100 PROTEIN (PTHR23155:SF1165) | antimicrobial response protein |
| Q2QZV8 | Q2QZV8_176_320 | Expressed protein | EXPRESSED PROTEIN (PTHR31713:SF10) | - |
| A0A0P0Y5Q8 | A0A0P0Y5Q8_99_222 | Os11g0691290 protein | OS12G0615100 PROTEIN (PTHR33491:SF32) | - |
| Q84ZI6 | Q84ZI6_430_646 | Os07g0129900 protein | OS07G0129900 PROTEIN (PTHR27007:SF41) | - |
| Q2R037 | Q2R037_2_321 | Expressed protein | SUPERKILLER COMPLEX PROTEIN 8 (PTHR44090:SF1) | - |
| Q53NL9 | Q53NL9_35_299 | Os11g0702100 protein | XYLANASE INHIBITOR PROTEIN 1 (PTHR45708:SF4) | - |
| A0A0P0WSE1 | A0A0P0WSE1_45_199 | Os06g0121000 protein | OS06G0121700 PROTEIN (PTHR36480:SF9) | - |
| Q2R077 | Q2R077_59_355 | Lipase family protein, expressed | LIPASE FAMILY PROTEIN, EXPRESSED (PTHR45856:SF8) | hydrolase |
| A3BFX5 | A3BFX5_1_144 | Os07g0112200 protein | - | - |
| Q5VQ96 | Q5VQ96_13_96_116_201_426_452, Q5VQ96_505_716 | Os06g0120200 protein | OS06G0120200 PROTEIN (PTHR31325:SF36) | - |
| Q2QZH9 | Q2QZH9_485_562 | NB-ARC domain containing protein, expressed | NB-ARC DOMAIN CONTAINING PROTEIN, EXPRESSED (PTHR23155:SF983) | antimicrobial response protein |
| A0A0N7KU10 | A0A0N7KU10_166_320 | Os12g0477000 protein | OS05G0492600 PROTEIN (PTHR23155:SF1226) | antimicrobial response protein |
| Q5Z8H5 | Q5Z8H5_47_172_318_329 | Peroxidase | PEROXIDASE (PTHR31517:SF34) | - |
| Q10MH8 | Q10MH8_189_424 | DEAD-box ATP-dependent RNA helicase 24 | ATP-DEPENDENT RNA HELICASE DDX42 (PTHR24031:SF125) | RNA helicase |
| Q7FAZ3 | Q7FAZ3_21_151 | G-type lectin S-receptor-like serine_threonine-protein kinase LECRK1 | G-TYPE LECTIN S-RECEPTOR-LIKE SERINE_THREONINE-PROTEIN KINASE LECRK1 (PTHR47976:SF108) | transmembrane signal receptor |
| Q7F8X6 | Q7F8X6_11_249_456_482 | Glycosyltransferase | GLYCOSYLTRANSFERASE (PTHR48047:SF79) | glycosyltransferase |
| Q6K785 | Q6K785_39_349 | Os02g0511400 protein | OS02G0511100 PROTEIN (PTHR43391:SF37) | dehydrogenase |
| A0A0P0W767 | A0A0P0W767_67_224 | Os04g0193950 protein | - | - |
| B9FR93 | B9FR93_19_115_131_191 | Os06g0123200 protein | OS06G0118900 PROTEIN (PTHR36480:SF3) | - |
| Q2R078 | Q2R078_99_147_165_422 | Leucine Rich Repeat family protein | - | - |
| A0A0P0Y553 | A0A0P0Y553_175_243 | Vacuolar protein sorting-associated protein | VACUOLAR-SORTING PROTEIN SNF8 (PTHR12806:SF0) | - |
| Q8GS08 | Q8GS08_36_187 | Dirigent protein | DIRIGENT PROTEIN (PTHR21495:SF196) | transporter |
| A0A0P0Y5N5 | A0A0P0Y5N5_500_684 | Os11g0694200 protein | OS11G0691500 PROTEIN (PTHR27005:SF162) | transmembrane signal receptor |
| Q0DIJ7 | Q0DIJ7_259_434 | Os05g0384300 protein | OS05G0384300 PROTEIN (PTHR47967:SF85) | - |
| A0A0P0X776 | A0A0P0X776_25_126 | Os07g0538300 protein | OS07G0538300 PROTEIN (PTHR32099:SF9) | - |
| Q8W3E8 | Q8W3E8_37_220 | COBRA-like protein 4 | COBRA-LIKE PROTEIN 4 (PTHR31673:SF3) | - |
| Q2QR00 | Q2QR00_166_319 | NB-ARC domain containing protein, expressed | OS05G0492600 PROTEIN (PTHR23155:SF1226) | antimicrobial response protein |
| A0A0P0Y5U5 | A0A0P0Y5U5_89_103_112_247 | Os11g0682501 protein | - | - |
| Q53NL2 | Q53NL2_33_279 | Os11g0701500 protein | XYLANASE INHIBITOR PROTEIN 1 (PTHR45708:SF4) | - |
| A0A0P0Y5B0 | A0A0P0Y5B0_9_159_324_373 | Os11g0681100 protein | OS11G0681100 PROTEIN (PTHR31325:SF44) | - |
| Q7XSK4 | Q7XSK4_63_393 | Os04g0191800 protein | OS04G0193300 PROTEIN (PTHR47993:SF382) | - |
| Q0DQB4 | Q0DQB4_57_217 | DNA polymerase kappa | DNA POLYMERASE KAPPA (PTHR11076:SF33) | DNA metabolism protein |

**S3 : CATH Superfamily and Fold Annotation for the Selected Domains**

**S3(a) Shortlisted mutation containing protein domains successfully mapped to CATH Superfamilies**

| **CATH Superfamily** | **Superfamily Name** | **# of Mutations** | **Domains Associated (Mutation Count)** |
| --- | --- | --- | --- |
| 3.40.50.300 | P-loop containing nucleotide triphosphate hydrolases | 10 | A0A0P0Y5U5_89_103_112_247(3), A0A0N7KU10_166_320(1), A0A0P0W767_67_224(1), Q10MH8_189_424(1), Q2QR00_166_319(1), Q2QV98_171_319(1), Q2QZS0_174_325(1), Q0IP79_169_317(1) |
| 3.80.10.10 | Ribonuclease Inhibitor | 9 | Q0IR17_45_199(1), Q0IR17_30_39_53_154(6), A0A0N7KU10_166_320(1), Q2R078_99_147_165_422(1) |
| 1.10.510.10 | Transferase(Phosphotransferase) domain 1 | 9 | A0A0P0Y5G5_8_175(2), C7J8J8_12_128(2), Q53QC7_513_710(2), A0A0P0Y5N5_500_684(1), Q84ZI6_430_646(1), Q6ZDZ0_482_491_500_559_585_693(1) |
| 3.20.20.80 | Glycosidases | 6 | Q53NL2_33_279(1), Q53NL3_32_285(1), Q53NL9_35_299(1), A0A0P0Y5Q7_33_298(1), O49826_30_284(1), Q0IQX8_32_277(1) |
| 3.60.21.10 | Metallo-dependent phosphatases | 5 | A0A0P0X1S7_7_245(2), Q0D921_167_606(2), A0A0P0X1N3_13_195_202_226(1) |
| 2.130.10.10 | YVTN repeat-like/Quinoprotein amine dehydrogenase | 3 | A0A5S6R7C8_49_268_285_318_337_379_392_409(1), Q0IR99_2_148_160_321(1), Q2R037_2_321(1) |
| 2.40.70.10 | Acid Proteases | 3 | Q0DIJ7_259_434(2), Q0JG81_31_201(1) |
| 3.30.200.20 | Phosphorylase Kinase; domain 1 | 3 | A0A0P0Y5N1_5_124(3) |
| 3.40.395.10 | Adenoviral Proteinase; Chain A | 3 | A0A0P0XSV6_275_288_423_521_531_662(1), Q2RBQ7_46_226(1), Q0IVA2_17_222(1) |
| 1.10.10.10 | "winged helix" repressor DNA binding domain | 2 | A0A0P0Y553_175_243(1), Q2QZH9_485_562(1) |
| 2.30.39.10 | Alpha-1-antitrypsin, domain 1 | 2 | C7IZM0_51_156_219_258(1), Q75H81_191_287_354_390(1) |
| 2.40.10.120 | Periplasmic serine endoprotease DegP-like | 2 | B9FA56_221_429(1), Q75GX1_220_429(1) |
| 2.60.40.630 | STAT transcription factor, DNA-binding domain | 2 | Q2QZV8_176_320(1), Q0IR75_175_322(1) |
| 3.30.559.10 | Chloramphenicol Acetyltransferase | 2 | A0A0P0VJK1_180_396(2) |
| 3.50.50.60 | FAD/NAD(P)-binding domain | 2 | Q0IMT5_12_102_160_234_341_418(1), Q2QP28_13_60_163_233_342_417(1) |
| 1.10.420.10 | Peroxidase, domain 2 | 1 | Q0DQB4_57_217(1) |
| 1.10.520.10 | Catalase-peroxidase | 1 | Q5Z8H5_47_172_318_329(1) |
| 1.20.1110.10 | Calcium-transporting ATPase, transmembrane domain | 1 | A0A0P0W110_218_425(1) |
| 1.20.120.1230 | Sucrose synthase | 1 | Q6K973_161_186_195_274(1) |
| 2.40.40.10 | Barwin-like endoglucanases | 1 | Q9FWT5_59_213(1) |
| 2.40.480.10 | Allene oxide cyclase-like | 1 | Q8GS08_36_187(1) |
| 2.60.120.330 | B-lactam Antibiotic, Isopenicillin N Synthase | 1 | Q67VR7_27_366(1) |
| 2.60.120.430 | Galactose-binding lectin | 1 | Q8H651_204_404(1) |
| 2.60.40.290 | Glucanase | 1 | Q8W3E8_37_220(1) |
| 2.90.10.10 | Agglutinin, subunit A | 1 | Q7FAZ3_21_151(1) |
| 3.30.2350.10 | Pseudouridine synthase | 1 | A0A0P0XHS6_50_353(1) |
| 3.30.420.10 | Ribonuclease H-like superfamily/Ribonuclease H | 1 | A3BFX5_1_144(1) |
| 3.30.430.20 | Gnk2 domain, C-X8-C-X2-C motif | 1 | A0A0P0X776_25_126(1) |
| 3.40.47.10 | Thiolase/Chalcone synthase | 1 | A0A0P0VR33_241_308_327_422(1) |
| 3.40.50.1820 | Alpha/Beta hydrolase fold, catalytic domain | 1 | Q2R077_59_355(1) |
| 3.40.50.2000 | Carbamoyl-phosphate synthase large chain | 1 | Q7F8X6_11_249_456_482(1) |
| 3.40.50.720 | NAD(P)-binding Rossmann-like Domain | 1 | Q6K785_39_349(1) |
| 3.40.50.880 | glutamine amidotransferase (GATase) domain | 1 | Q0JH25_516_793(1) |

**S3 (b) Shortlisted mutation containing protein domains successfully mapped to CATH Folds**

| **CATH Fold** | **Number of Mutations** | **Domains Associated (Mutation Count)** |
| --- | --- | --- |
| 2.60.40 | 12 | A0A0P0WSE1(10), B9FR93(2) |
| 2.120.10 | 2 | Q0JEW8(1), Q7XSK4(1) |
| 1.25.40 | 1 | A0A0N7KIR5(1) |
| 3.80.10 | 2 | C7J8A2(1), Q69X07(1) |
| 1.20.1050 | 1 | Q5VQ96(1) |
| 1.20.190 | 1 | A0A0P0Y5B0(1) |
| 2.40.70 | 1 | Q2QZP4(1) |
| 2.60.120 | 1 | A0A0P0Y5Q8(1) |

**S4 InterPro and Pfam annotation**

**S4 Shortlisted mutation containing proteins domains mapped to Interpro and Pfam**

| **Domain** | **InterPro** | **Name** | **Pfam** | **Name.1** | **Mutation Count** |
| --- | --- | --- | --- | --- | --- |
| A0A0N7KIR5_4_111 | IPR033133 | Pumilio homology domain | PS50303 | Pumilio homology domain (PUM-HD) profile | 1 |
| A0A0N7KU10_166_320 | IPR002182 | NB-ARC domain | PF00931 | NB-ARC domain | 1 |
| A0A0P0VJK1_180_396 | IPR023213 | Chloramphenicol acetyltransferase-like domain superfamily | PF00198 | 2-oxoacid dehydrogenases acyltransferase (catalytic domain) | 2 |
| A0A0P0VR33_241_308_327_422 | IPR012392 | Very-long-chain 3-ketoacyl-CoA synthase | PF08541 | 3-Oxoacyl-[acyl-carrier-protein (ACP)] synthase III C terminal | 1 |
| A0A0P0W110_218_425 | IPR001757 | P-type ATPase | PF00689 | Cation transporting ATPase, C-terminus | 1 |
| A0A0P0W767_67_224 | IPR027417 | P-loop containing nucleoside triphosphate hydrolase | PF00931 | NB-ARC domain | 1 |
| A0A0P0X1N3_13_195_202_226 | IPR029052 | Metallo-dependent phosphatase-like | PF00149 | Calcineurin-like phosphoesterase | 1 |
| A0A0P0X1S7_7_245 | IPR029052 | Metallo-dependent phosphatase-like | PF00149 | Calcineurin-like phosphoesterase | 2 |
| A0A0P0X776_25_126 | IPR002902 | Gnk2-homologous domain | PF01657 | Salt stress response/antifungal | 1 |
| A0A0P0XHS6_50_353 | IPR050188 | RluA Pseudouridine Synthase | PF00849 | RNA pseudouridylate synthase | 1 |
| A0A0P0XSV6_275_288_423_521_531_662 | IPR038765 | Papain-like cysteine peptidase superfamily | | | 1 |
| A0A0P0Y553_175_243 | IPR036388 | Winged helix-like DNA-binding domain superfamily | PF04157 | EAP30/Vps36 family | 1 |
| A0A0P0Y5B0_9_159_324_373 | IPR025315 | Domain of unknown function DUF4220 | PF13968 | Domain of unknown function (DUF4220) | 1 |
| A0A0P0Y5G5_8_175 | IPR045274 | WALL-ASSOCIATED RECEPTOR Receptor-like kinase WAK-likeKINASE-LIKE 21 | | | 2 |
| A0A0P0Y5N1_5_124 | IPR045274 | WALL-ASSOCIATED RECEPTOR KINASE-LIKE 21 | PF07714 | Protein tyrosine and serine/threonine kinase | 3 |
| A0A0P0Y5N5_500_684 | IPR045274 | WALL-ASSOCIATED RECEPTOR KINASE-LIKE 21 | PF00069 | Protein kinase domain | 1 |
| A0A0P0Y5Q7_33_298 | IPR050542 | Glycosyl Hydrolase 18 Family Chitinases | PF00704 | Glycosyl hydrolases family 18 | 1 |
| A0A0P0Y5U5_89_103_112_247 | IPR027417 | P-loop containing nucleoside triphosphate hydrolase | PF00931 | NB-ARC Domain | 3 |
| A0A5S6R7C8_49_268_285_318_337_379_392_409 | IPR016902 | Vacuolar protein sorting-associated protein Vps41 | PF23411 | Vacuolar protein sorting-associated protein Vps41 | 1 |
| A3BFX5_1_144 | IPR051132 | 3'-5' Exonuclease domain-containing protein | PF01612 | 3'-5' exonuclease | 1 |
| B9FA56_221_429 |  |  | PF13365 | Trypsin-like peptidase domain | 1 |
| C7IZM0_51_156_219_258 | IPR023796 | Serine Protease Inhibitor | PF00079 | Serine Protease Inhibitor | 1 |
| C7J8J8_12_128 | PR052451 | Serine Threonine Kinase | | | 2 |
| O49826_30_284 | IPR050542 | Glycosyl Hydrolase 18 Family Chitinases | PF00704 | Glycosyl hydrolases family 18 | 1 |
| Q0D921_167_606 | IPR029052 | Metallo-dependent phosphatase-like | PF00149 | Calcineurin-like phosphoesterase | 2 |
| Q0DIJ7_259_434 | IPR021109 | Acid Protease | PF14541 | Xylanase inhibitor C-terminal | 2 |
| Q0IMT5_12_102_160_234_341_418 | IPR045170 | PEROXISOMAL SARCOSINE OXIDASE | PF01266 | FAD dependent oxidoreductase | 1 |
| Q0IP79_169_317 | IPR027417 | P-loop containing nucleoside triphosphate hydrolases | PF00931 | NB-ARC domain | 1 |
| Q0IQX8_32_277 | PR050542 | Glycosyl Hydrolase 18 Family Chitinases | PF00704 | Glycosyl hydrolases family 18 | 1 |
| Q0IR17_30_39_53_154 | IPR032675 | Ribonuclease Inhibitor | PF23598 | Leucine-rich repeat region | 6 |
| Q0IR17_45_199 | IPR032675 | Ribonuclease Inhibitor | PF23598 | Leucine-rich repeat region | 1 |
| Q0IR63_201_321 | IPR011990 | Tetratricopeptide repeat domain | PF01535 | PPR- Repeat Family | 1 |
| Q0IR75_175_322 | IPR046831 | Calmodulin binding protein-like N-terminal domain | PF07887 | Calmodulin binding protein-like N-terminal domain | 1 |
| Q0IR99_2_148_160_321 | IPR015943 | YVTN repeat-like/Quinoprotein amine dehydrogenase | PF00400 | WD domain, G-beta repeat | 1 |
| Q0IVA2_17_222 | IPR044613 | SENTRIN-SPECIFIC PROTEASE 8 | PF02902 | Ulp1 protease family, C-terminal catalytic domain | 1 |
| Q0JEW8_64_395 | IPR017451 | F-Box Interaction domain | PF08268 | F-box-associated | 1 |
| Q0JG81_31_201 | IPR021109 | Acid Protease | PF14543 | Xylanase inhibitor N-terminal | 1 |
| Q0JH25_516_793 | IPR027417 | P-loop containing nucleoside triphosphate hydrolase | PF00271 | Helicase conserved C-terminal domain | 1 |
| Q10MH8_189_424 | PR027417 | P-loop containing nucleoside triphosphate hydrolase | PF00270 | DEAD/DEAH box helicase | 1 |
| Q2QP28_13_60_163_233_342_417 | IPR045170 | PEROXISOMAL SARCOSINE OXIDASE | PF01266 | FAD dependent oxidoreductase | 1 |
| Q2QR00_166_319 | IPR027417 | P-loop containing nucleoside triphosphate hydrolase | PF00931 | NB-ARC domain | 1 |
| Q2QV98_171_319 | IPR027417 | P-loop containing nucleoside triphosphate hydrolase | PF00931 | NB-ARC domain | 1 |
| Q2QZH9_485_562 | IPR036388 | Winged helix-like DNA-binding domain superfamily/Winged helix DNA-binding domain | PF23559 | Disease resistance protein Winged helix domain | 1 |
| Q2QZP4_23_137 | IPR054722 | Pol polyprotein, beta-barrel domain | PF22936 | Pol polyprotein, beta-barrel domain | 1 |
| Q2QZS0_174_325 | :IPR027417 | P-loop containing nucleoside triphosphate hydrolase | PF00931 | NB-ARC domain | 1 |
| Q2QZV8_176_320 | IPR046831 | Calmodulin binding protein-like N-terminal domain | PF07887 | Calmodulin binding protein-like N-terminal domain | 1 |
| Q2R037_2_321 | IPR015943 | YVTN repeat-like/Quinoprotein amine dehydrogenase | PF23414 | Echinoderm microtubule-associated protein second beta-propeller | 1 |
| Q2R077_59_355 | IPR029058 | Alpha/Beta Hydrolase | PF01764 | Lipase_3 | 1 |
| Q2R078_99_147_165_422 | IPR032675 | Ribonuclease Inhibitor | PF23598 | Leucine-rich repeat region | 1 |
| Q2RBQ7_46_226 | IPR032675 | Ribonuclease Inhibitor | PF23598 | Leucine-rich repeat region | 1 |
| Q53NL2_33_279 | : IPR050542 | Glycosyl Hydrolase 18 Family Chitinases | PF00704 | Glycosyl hydrolases family 18 | 1 |
| Q53NL3_32_285 | : IPR050542 | Glycosyl Hydrolase 18 Family Chitinases | PF00704 | Glycosyl hydrolases family 18 | 1 |
| Q53NL9_35_299 | IPR050542 | Glycosyl Hydrolase 18 Family Chitinases | PF00704 | Glycosyl hydrolases family 18 | 1 |
| Q53QC7_513_710 | IPR045274 | WALL-ASSOCIATED RECEPTOR KINASE-LIKE 21 | PF00069 | Protein kinase domain | 2 |
| Q5VQ96_13_96_116_201_426_452 | PR025315 | Domain of unknown function DUF4220 | PF13968 | Domain of unknown function DUF4220 | 1 |
| Q5VQ96_505_716 |  |  | PF04578 | Protein of unknown function, DUF594 | 1 |
| Q5Z8H5_47_172_318_329 | IPR002016 | Plant heme peroxidase family profile | PF00141 | peroxidase | 1 |
| Q67VR7_27_366 | IPR050231 | Iron/ascorbate-dependent oxidoreductase | PF14226 | non-haem dioxygenase in morphine synthesis N-terminal | 1 |
| Q69X07_347_464 | IPR032675 | Ribonuclease Inhibitor | PF23598 | Leucine-rich repeat region | 1 |
| Q6K785_39_349 |  |  | PF00106 | Short Chain Dehydrogenase | 1 |
| Q6K973_161_186_195_274 | IPR012820 | Sucrose synthase, plant | PF24862 | Sucrose synthase EPBD domain | 1 |
| Q6ZDZ0_482_491_500_559_585_693 | IPR046959 | LEUCINE-RICH REPEAT RECEPTOR-LIKE PROTEIN KINASE PXC1 | PF07714 | Protein tyrosine and serine/threonine kinase | 1 |
| Q75GX1_220_429 |  |  | PF13365 | Trypsin-like peptidase domain | 1 |
| Q75H81_191_287_354_390 | IPR000215 | SERINE PROTEASE INHIBITOR, SERPIN | PF00079 | Serine Protease Inhibitor | 1 |
| Q7F8X6_11_249_456_482 | IPR002213 | UDP-glucuronosyl/UDP-glucosyltransferase | PF00201 | UDP-glucoronosyl and UDP-glucosyl transferase | 1 |
| Q7FAZ3_21_151 | IPR036426 | Bulb-type lectin domain | PF01453 | D-mannose binding lectin | 1 |
| Q7XSK4_63_393 | IPR017451 | F-Box Interaction domain | PF08268 | F-box-associated | 1 |
| Q84ZI6_430_646 | IPR050528 | L-type Lectin-Domain Containing Receptor Kinases | PF00069 | Protein kinase domain | 1 |
| Q8GS08_36_187 | IPR044859 | Allene oxide cyclase-like | PF03018 | Dirigent Lke Protein | 1 |
| Q8H651_204_404 | IPR024788 | Malectin like domain | PF12819 | Malectin like domain | 1 |
| Q8W3E8_37_220 | IPR006918 | COBRA Protein | PF04833 | COBRA-like Protein | 1 |
| Q9FWT5_59_213 | IPR039271 | Kiwellin-like | PF24300 | Kiwellin-1 | 1 |

**S5: Functional enrichment analysis of the mutation associated protein coding genes**

| **source** | **term_name** | **term_id** | **adjusted_p_value (<0.05=significant)** | **Genes covered out of 51** |
| --- | --- | --- | --- | --- |
| GO:MF | chitinase activity | GO:0004568 | 0.00425813 | 3 |
| GO:MF | catalytic activity | GO:0003824 | 0.0052059 | 26 |
| GO:MF | anion binding | GO:0043168 | 0.0052059 | 14 |
| GO:MF | heterocyclic compound binding | GO:1901363 | 0.0052059 | 14 |
| GO:MF | ADP binding | GO:0043531 | 0.0052059 | 5 |
| GO:MF | adenyl ribonucleotide binding | GO:0032559 | 0.00641735 | 12 |
| GO:MF | adenyl nucleotide binding | GO:0030554 | 0.00715083 | 12 |
| GO:MF | nucleoside phosphate binding | GO:1901265 | 0.00715083 | 13 |
| GO:MF | nucleotide binding | GO:0000166 | 0.00715083 | 13 |
| GO:MF | purine ribonucleotide binding | GO:0032555 | 0.0088097 | 12 |
| GO:MF | ribonucleotide binding | GO:0032553 | 0.0088183 | 12 |
| GO:MF | carbohydrate derivative binding | GO:0097367 | 0.00883891 | 12 |
| GO:MF | purine nucleotide binding | GO:0017076 | 0.00993183 | 12 |
| GO:MF | molecular_function | GO:0003674 | 0.011224477352528614 | 35 |
| GO:MF | carbohydrate binding | GO:0030246 | 0.012386051876243323 | 4 |
| GO:MF | ATP binding | GO:0005524 | 0.01842372 | 10 |
| GO:MF | S-succinyltransferase activity | GO:0016751 | 0.01842372 | 1 |
| GO:MF | succinyltransferase activity | GO:0016748 | 0.01842372 | 1 |
| GO:MF | leucocyanidin oxygenase activity | GO:0050589 | 0.01842372 | 1 |
| GO:MF | phosphoribosylformylglycinamidine synthase activity | GO:0004642 | 0.01842372 | 1 |
| GO:MF | dihydrolipoyllysine-residue succinyltransferase activity | GO:0004149 | 0.01842372 | 1 |
| GO:MF | hydrolase activity, acting on glycosyl bonds | GO:0016798 | 0.022881290874524042 | 4 |
| GO:MF | ion binding | GO:0043167 | 0.022881290874524042 | 17 |
| GO:MF | sarcosine oxidase activity | GO:0008115 | 0.02416564 | 1 |
| GO:MF | small molecule binding | GO:0036094 | 0.027002601348211884 | 17 |
| GO:MF | purine ribonucleoside triphosphate binding | GO:0035639 | 0.027123274528706207 | 10 |
| GO:MF | deNEDDylase activity | GO:0019784 | 0.03575507 | 1 |
| GO:MF | transmembrane receptor protein kinase activity | GO:0019199 | 0.03889095 | 2 |
| GO:MF | transmembrane signaling receptor activity | GO:0004888 | 0.04210329 | 2 |
| GO:BP | xylan catabolic process | GO:0045493 | 3.8277E-06 | 4 |
| GO:BP | hemicellulose catabolic process | GO:2000895 | 3.8277E-06 | 4 |
| GO:BP | cell wall polysaccharide catabolic process | GO:0044347 | 3.8277E-06 | 4 |
| GO:BP | cell wall macromolecule catabolic process | GO:0016998 | 2.263E-05 | 4 |
| GO:BP | cell wall polysaccharide metabolic process | GO:0010383 | 0.00031163 | 5 |
| GO:BP | cell wall macromolecule metabolic process | GO:0044036 | 0.00046033 | 5 |
| GO:BP | xylan metabolic process | GO:0045491 | 0.00103739 | 4 |
| GO:BP | defense response | GO:0006952 | 0.00148032 | 8 |
| GO:BP | biological_process | GO:0008150 | 0.00235589 | 35 |
| GO:BP | response to stress | GO:0006950 | 0.00235589 | 11 |
| GO:BP | hemicellulose metabolic process | GO:0010410 | 0.00235589 | 4 |
| GO:BP | metabolic process | GO:0008152 | 0.00357121 | 28 |
| GO:BP | polysaccharide catabolic process | GO:0000272 | 0.00357121 | 4 |
| GO:BP | response to external stimulus | GO:0009605 | 0.00357121 | 7 |
| GO:BP | catabolic process | GO:0009056 | 0.00498497 | 9 |
| GO:BP | polysaccharide metabolic process | GO:0005976 | 0.00741313 | 5 |
| GO:BP | cell wall organization or biogenesis | GO:0071554 | 0.00741313 | 5 |
| GO:BP | response to external biotic stimulus | GO:0043207 | 0.011815073636162224 | 6 |
| GO:BP | response to other organism | GO:0051707 | 0.011815073636162224 | 6 |
| GO:BP | carbohydrate catabolic process | GO:0016052 | 0.01196046 | 4 |
| GO:BP | biological process involved in interspecies interaction between organisms | GO:0044419 | 0.012338962796967808 | 6 |
| GO:BP | response to biotic stimulus | GO:0009607 | 0.012338962796967808 | 6 |
| GO:BP | primary metabolic process | GO:0044238 | 0.012478980396463214 | 24 |
| GO:BP | defense response to other organism | GO:0098542 | 0.016172498318761122 | 5 |
| GO:BP | macromolecule catabolic process | GO:0009057 | 0.018669567747889013 | 6 |
| GO:BP | carbohydrate metabolic process | GO:0005975 | 0.029193059556917013 | 6 |
| GO:BP | L-lysine catabolic process to acetyl-CoA | GO:0019474 | 0.03886096 | 1 |
| GO:BP | establishment of protein localization to vacuole | GO:0072666 | 0.03886096 | 2 |
| GO:BP | response to stimulus | GO:0050896 | 0.03886096 | 12 |
| GO:BP | protein localization to vacuole | GO:0072665 | 0.03886096 | 2 |
| GO:BP | positive regulation of wax biosynthetic process | GO:1904278 | 0.03886096 | 1 |
| GO:BP | regulation of wax biosynthetic process | GO:1904276 | 0.03886096 | 1 |
| GO:BP | lysine catabolic process | GO:0006554 | 0.03886096 | 1 |
| GO:BP | L-lysine catabolic process to acetyl-CoA via saccharopine | GO:0033512 | 0.03886096 | 1 |
| GO:BP | L-lysine metabolic process | GO:0046440 | 0.03886096 | 1 |
| GO:BP | L-lysine catabolic process | GO:0019477 | 0.03886096 | 1 |
| GO:BP | cellular process | GO:0009987 | 0.04570732 | 29 |
| GO:BP | endosomal vesicle fusion | GO:0034058 | 0.049056061846491895 | 1 |
| GO:CC | extracellular region | GO:0005576 | 0.00033389 | 8 |
| KEGG | Amino sugar and nucleotide sugar metabolism | KEGG:00520 | 0.00983869 | 3 |
| KEGG | KEGG root term | KEGG:00000 | 0.04144336 | 11 |
| KEGG | Metabolic pathways | KEGG:01100 | 0.04144336 | 7 |

Note: MF: Molecular Function, BP: Biological Process, CC: cellular component

**S6 Functional Site Analysis**

**Rice functional site residues that lie within 5 angstroms to the mutated residues**

| **Domain** | **Substitution** | **Grantham** | **Functional_Pocket** | **Distance_pocket** | **Functional_allosteric site** | **Distance_allosteric** | **Functional_PTM** | **Distance_PTM** | **Functional_Conserved Sites** | **Distance_Conserved Sites** | **Functional_small_molecule_binding** | **Distance_small moleculae binding** | **Functional_ProteinProtein interface** | **Distance_ProteinProtein interface** |
| --- | --- | --- | --- | --- | --- | --- | --- | --- | --- | --- | --- | --- | --- | --- |
| A0A0N7KU10_166_320 | S263L | 145 |  |  |  |  |  |  | 259 | 3.1 |  |  |  |  |
| A0A0P0VJK1_180_396 | L210R | 102 |  |  |  |  |  |  | 227 | 3.5 | 227 | 3.5 |  |  |
| A0A0P0VR33_241_308_327_422 | A366T | 58 |  |  |  |  |  |  |  |  |  |  | 367 | 1.3 |
| A0A0P0XHS6_50_353 | T226I | 89 |  |  | 225 | 1.3 |  |  |  |  |  |  |  |  |
| A0A0P0Y5G5_8_175 | R130C | 180 |  |  | 133 | 3.2 |  |  |  |  |  |  |  |  |
| A0A0P0Y5N1_5_124 | H85L | 99 |  |  |  |  |  |  |  |  | 110 | 4.8 | 84 | 1.3 |
| A0A0P0Y5N1_5_124 | G119E | 98 |  |  |  |  |  |  |  |  | 120 | 1.3 | 120 | 1.3 |
| A0A0P0Y5N1_5_124 | K29E | 56 |  |  |  |  |  |  |  |  | 30 | 1.3 |  |  |
| A0A0P0Y5N5_500_684 | H527Y | 83 |  |  |  |  | 521 | 5.1 | 582 | 2.8 | 531 | 2.9 | 531 | 2.9 |
| A0A0P0Y5Q8_99_222 | T220A | 58 |  |  |  |  |  |  |  |  |  |  | 219 | 1.3 |
| A0A0P0Y5U5_89_103_112_247 | N220Y | 143 |  |  | 114 | 3.1 |  |  |  |  |  |  |  |  |
| A0A0P0Y5U5_89_103_112_247 | S234R | 110 |  |  | 236 | 2.6 |  |  |  |  | 238 | 4.5 | 233 | 1.3 |
| A0A0P0Y5U5_89_103_112_247 | A232S | 99 |  |  |  |  |  |  |  |  | 231 | 1.3 | 233 | 1.3 |
| A3BFX5_1_144 | H25N | 68 |  |  |  |  |  |  |  |  |  |  | 23 | 3.2 |
| B9FR93_19_115_131_191 | F184S | 155 |  |  |  |  |  |  | 165 | 5.1 |  |  | 183 | 1.3 |
| B9FR93_19_115_131_191 | C164S | 112 |  |  |  |  |  |  | 165 | 1.3 |  |  |  |  |
| O49826_30_284 | K163N | 94 |  |  |  |  |  |  |  |  | 118B | 3.5 |  |  |
| Q0DIJ7_259_434 | M287T | 81 |  |  |  |  |  |  | 320 | 3.8 |  |  |  |  |
| Q0IR63_201_321 | G311S | 56 |  |  |  |  |  |  | 310 | 1.3 |  |  | 310 | 1.3 |
| Q0IR75_175_322 | A201T | 58 |  |  |  |  |  |  | 194 | 5.1 |  |  |  |  |
| Q0IVA2_17_222 | S69P | 74 |  |  |  |  | 143 | 3.5 |  |  |  |  | 67 | 3.2 |
| Q10MH8_189_424 | P217S | 74 |  |  |  |  |  |  | 218 | 1.3 |  |  |  |  |
| Q2QR00_166_319 | T305S | 58 |  |  |  |  |  |  |  |  |  |  | 303 | 3.8 |
| Q2QV98_171_319 | D183V | 152 |  |  |  |  |  |  |  |  |  |  | 180 | 3.3 |
| Q2QZH9_485_562 | S501T | 58 |  |  |  |  |  |  |  |  |  |  | 502 | 1.3 |
| Q2QZS0_174_325 | H321Y | 83 |  |  |  |  |  |  |  |  |  |  | 322 | 1.3 |
| Q2R078_99_147_165_422 | Q104K | 53 |  |  |  |  |  |  |  |  |  |  | 107 | 1.3 |
| Q2RBQ7_46_226 | S69P | 74 |  |  |  |  | 143 | 3.5 |  |  |  |  | 67 | 3.2 |
| Q53NL2_33_279 | R227W | 101 |  |  |  |  |  |  |  |  | 263 | 4.9 |  |  |
| Q53QC7_513_710 | C591S | 112 |  |  |  |  |  |  |  |  | 557 | 4.1 | 592 | 1.3 |
| Q5VQ96_505_716 | K638T | 78 |  |  |  |  |  |  |  |  |  |  | 515 | 4.3 |
| Q5Z8H5_47_172_318_329 | A116T | 58 |  |  |  |  |  |  |  |  | 112 | 3.1 | 142 | 3.3 |
| Q6K785_39_349 | G191A | 60 | 192 | 1.3 |  |  |  |  |  |  | 192 | 1.3 | 192 | 1.3 |
| Q7F8X6_11_249_456_482 | H21D | 81 | 20 | 1.3 |  |  |  |  |  |  |  |  | 22 | 1.3 |
| Q7FAZ3_21_151 | R129C | 180 |  |  |  |  |  |  |  |  |  |  | 131 | 3.1 |
| Q7XSK4_63_393 | A254G | 60 |  |  | 232 | 3.9 |  |  |  |  |  |  |  |  |
| Q8GS08_36_187 | P61L | 98 | 72 | 4.2 |  |  |  |  |  |  |  |  | 60 | 1.3 |
| Q8H651_204_404 | M206T | 81 |  |  |  |  |  |  |  |  |  |  | 205 | 1.3 |
| Q9FWT5_59_213 | D175G | 94 | 176 | 1.3 |  |  |  |  |  |  |  |  |  |  |

Note: for the analysis, we have combined P2Rank predicted pockets and AlphaFill predicted small molecule binding sites, as ligand binding sites in the main text.

**S7. Interaction partners of Rice domains**

| **Protein domain ID** | **Protein name in UniProt** | **Predicted interactor** | **Interactor name in UniProt** | **ipTM** | **pTM** | **Average plDDT** | **PRODIGY** | **PIZSA score** |
| --- | --- | --- | --- | --- | --- | --- | --- | --- |
| A0A0P0VR33_241_308_327_422 | 3-ketoacyl-CoA synthase | Q2QMH0 | Os12g0604800 protein | 0.16 | 0.55 | 79.12 | -10.1 | 1.381 |
| A0A0P0Y5N1_5_124 | Os11g0690600 protein | A0A0P0V1T6 | Os01g0314700 protein | 0.72 | 0.69 | 69.78 | -11.3 | 1.8 |
| A0A0P0Y5N5_500_684 | Os11g0694200 protein | Q8RUE8 | serine/threonine protein kinase | 0.78 | 0.51 | 70.61 | -8.4 | 1.6 |
| A0A0P0Y5Q8_99_222 | Os11g0691290 protein | A0A0P0V1T6 | Os01g0314700 protein | 0.7 | 0.6 | 71.92 | -8.5 | 2.19 |
| A3BFX5_1_144 | Os07g0112200 protein | EXO1_Q60GC1 | Exonuclease 1 | 0.36 | 0.39 | 50.99 | -11.5 | 1.59 |
| Q2QR00_166_319 | NB-ARC domain containing protein | Q10EP9 | Aurora kinase | 0.727 | 0.57 | 66.98 | -16.5 | 1.118 |
| Q2QV98_171_319 | NB-ARC domain containing protein | Q10EP9 | Aurora kinase | 0.716 | 0.6 | 66.48 | -15.3 | 0.195 |
| Q2QZH9_485_562 | NB-ARC domain containing protein | Q10EP9 | Aurora kinase | 0.22 | 0.47 | 61.21 | -14.6 | 0.05 |
| Q2QZS0_174_325 | NB-ARC domain containing protein | Q10EP9 | Aurora kinase | 0.26 | 0.64 | 69.75 | -15.3 | 0.71 |
| Q2R078_99_147_165_422 | Leucine Rich Repeat family protein | Q10EP9 | Aurora kinase | 0.61 | 0.53 | 76.28 | -12.1 | 1.62 |
| Q2RBQ7_46_226 | Os11g0102700 protein | RUB1 | Ubiquitin-NEDD8-like protein RUB1 | 0.6 | 0.7 | 80.63 | -15.2 | 1.34 |
| Q5VQ96_505_716 | Os06g0120200 protein | C7J1U6 | Os04g0589400 protein | 0.15 | 0.48 | 54.43 | -5.4 | 2.011 |
| Q5Z8H5_47_172_318_329 | Peroxidase | CAD6 | cinnamyl-alcohol dehydrogenase | 0.74 | 0.55 | 83.4 | -11.4 | 1.73 |
| Q6K785_39_349 | Os02g0511400 protein | Q2QRX6 | Putative bifunctional dihydrofolate reductase-thymidylate synthase | 0.73 | 0.55 | 74.4 | -14 | 0.24 |
| Q7F8X6_11_249_456_482 | Glycosyltransferase | A0A0P0UYV2 | Os01g0172600 protein | 0.71 | 0.69 | 80.22 | -7.3 | 0.136 |
| Q7FAZ3_21_151 | G-type lectin S-receptor-like serine/threonine-protein kinase | Q0JF21 | Lectin | 0.2 | 0.46 | 75.58 | -10.5 | 1.118 |
| Q8GS08_36_187 | Dirigent protein | P0552F09102 | Os07g0616900 protein | 0.25 | 0.41 | 61.45 | -6.9 | 0.08 |
| Q8H651_204_404 | Os06g0126250 protein | A0A0P0V1T6 | Os01g0314700 protein | 0.72 | 0.4 | 70.42 | -22.5 | 1.72 |

**S8. Impact of mutations lying beyond 5 angstroms to a known or predicted functional site**

| **Protein (UniProt ID)** | **Mutation** | **Trait** | **Grantham Score** | **MutPred2 Score** | **FoldXDelta\Delta G(kcal/mol)** |
| --- | --- | --- | --- | --- | --- |
| A0A0N7KIR5 | A71T | Brown Plant Hopper Resistance | 58 | 0.159 | 0.325995 |
| A0A0P0VJK1 | L275P | Salt Stress (Seedling No.) | 102 | 0.164 | 6.11294 |
| A0A0P0W110 | T243I | Yellow Mottle Virus Resistance | 89 | 0.331 | -1.7082 |
| A0A0P0W767 | H81D | Brown Plant Hopper Resistance | 81 | 0.238 | -0.61256 |
| A0A0P0WSE1 | P121R | Brown Plant Hopper Resistance | 145 | 0.653 | -0.47261 |
| A0A0P0WSE1 | N67Y | Brown Plant Hopper Resistance | 143 | 0.605 | -1.85872 |
| A0A0P0WSE1 | L98R | Brown Plant Hopper Resistance | 126 | 0.561 | 0.383274 |
| A0A0P0WSE1 | T69I | Brown Plant Hopper Resistance | 103 | 0.558 | -2.34218 |
| A0A0P0WSE1 | A56D | Brown Plant Hopper Resistance | 102 | 0.472 | -0.24454 |
| A0A0P0WSE1 | E54K | Brown Plant Hopper Resistance | 94 | 0.355 | -0.67859 |
| A0A0P0WSE1 | N125K | Brown Plant Hopper Resistance | 89 | 0.339 | -0.81453 |
| A0A0P0WSE1 | Q122K | Brown Plant Hopper Resistance | 64 | 0.193 | -0.08128 |
| A0A0P0WSE1 | S106L | Brown Plant Hopper Resistance | 56 | 0.136 | -1.5204 |
| A0A0P0WSE1 | A112V | Brown Plant Hopper Resistance | 53 | 0.104 | 0.81335 |
| A0A0P0X1N3 | L187G | Sheath Blight Disease Resistance | 138 | 0.909 | 5.65931 |
| A0A0P0X1S7 | N187G | Sheath Blight Disease Resistance | 81 | 0.624 | 0.86878 |
| A0A0P0X1S7 | T247M | Sheath Blight Disease Resistance | 80 | 0.461 | -2.41064 |
| A0A0P0X776 | E42G | Anaerobic Response Index | 127 | 0.33 | 1.39976 |
| A0A0P0XSV6 | R542C | Sheath Blight Disease Resistance | 180 | 0.211 | 2.0554 |
| A0A0P0Y553 | S202L | Bacterial Blight Resistance | 145 | 0.272 | 0.55094 |
| A0A0P0Y5B0 | W120L | Bacterial Blight Resistance | 56 | 0.853 | 0.593676 |
| A0A0P0Y5G5 | E116K | Bacterial Blight Resistance | 180 | 0.702 | -0.28159 |
| A0A0P0Y5Q7 | A179T | Bacterial Blight Resistance | 58 | 0.652 | -0.40174 |
| A0A5S6R7C8 | K138N | Brown Plant Hopper Resistance | 94 | 0.282 | 0.122465 |
| B9FA56 | A304T | Anaerobic Response Index | 58 | 0.06 | -0.01698 |
| C7IZM0 | D236R | Sheath Blight Disease Resistance | 96 | 0.827 | -1.04472 |
| C7J8J8 | L83R | Bacterial Blight Resistance | 102 | 0.686 | 3.10074 |
| C7J8J8 | N41T | Bacterial Blight Resistance | 65 | 0.235 | 0.358981 |
| Q0D921 | M242T | Sheath Blight Disease Resistance | 81 | 0.745 | 3.45392 |
| Q0D921 | D187G | Sheath Blight Disease Resistance | 94 | 0.7 | 2.23489 |
| Q0DIJ7 | G298A | Salinity Tolerance (Survival Days) | 81 | 0.149 | -0.93975 |
| Q0DQB4 | W58T | Yellow Mottle Virus Resistance | 128 | 0.896 | 4.42785 |
| Q0IMT5 | K210N | Total Lesion Length (Sheath Blight) | 94 | 0.476 | 3.15698 |
| Q0IP79 | D183V | Sheath Blight Disease Resistance | 152 | 0.424 | -0.19609 |
| Q0IQX8 | R227W | Bacterial Blight Resistance | 101 | 0.478 | -0.62639 |
| Q0IR17 | L129G | Bacterial Blight Resistance | 96 | 0.901 | 5.97697 |
| Q0IR17 | R57A | Bacterial Blight Resistance | 134 | 0.804 | 0.613877 |
| Q0IR17 | D82K | Bacterial Blight Resistance | 101 | 0.724 | 0.922284 |
| Q0IR17 | I103T | Bacterial Blight Resistance | 138 | 0.538 | 1.81055 |
| Q0IR17 | I128E | Bacterial Blight Resistance | 89 | 0.48 | 1.43525 |
| Q0IR17 | F73A | Bacterial Blight Resistance | 112 | 0.27 | 3.70193 |
| Q0IR17 | L116A | Bacterial Blight Resistance | 113 | 0.752 | 4.3049 |
| Q0IR99 | S59A | Bacterial Blight Resistance | 99 | 0.135 | 0.574896 |
| Q0JEW8 | A254G | Brown Plant Hopper Resistance | 60 | 0.438 | 1.48288 |
| Q0JG81 | N52K | Sheath Blight Disease Resistance | 94 | 0.212 | 0.508609 |
| Q0JH25 | L793S | Anaerobic Response Index | 145 | 0.822 | 0.095048 |
| Q2QP28 | K210N | Total Lesion Length (Sheath Blight) | 94 | 0.538 | 2.35885 |
| Q2QZP4 | R49C | Bacterial Blight Resistance | 180 | 0.291 | 0.681219 |
| Q2QZV8 | A201T | Yellow Mottle Virus Resistance | 58 | 0.206 | 0.25656 |
| Q2R037 | S59A | Bacterial Blight Resistance | 99 | 0.135 | 0.173749 |
| Q2R077 | P85S | Yellow Mottle Virus Resistance | 74 | 0.579 | 1.31264 |
| Q53NL3 | K163N | Bacterial Blight Resistance | 94 | 0.747 | 0.395013 |
| Q53NL9 | A179T | Bacterial Blight Resistance | 58 | 0.652 | -0.39452 |
| Q53QC7 | N686Y | Bacterial Blight Resistance | 112 | 0.381 | 0.043197 |
| Q5VQ96 | A185T | Brown Plant Hopper Resistance | 58 | 0.416 | -0.13542 |
| Q67VR7 | R302C | Salinity Tolerance (Survival Days) | 180 | 0.315 | 2.4895 |
| Q69X07 | G423R | Nitrogen Sensitivity | 125 | 0.217 | 2.35332 |
| Q6K973 | N224H | Total Lesion Length (Sheath Blight) | 68 | 0.184 | 0.392754 |
| Q6ZDZ0 | R672W | Sheath Blight Disease Resistance | 101 | 0.344 | -0.78911 |
| Q75GX1 | A304T | Anaerobic Response Index | 58 | 0.06 | 0.013351 |
| Q75H81 | G236R | Sheath Blight Disease Resistance | 125 | 0.226 | -0.88893 |
| Q84ZI6 | A542T | Blast Disease Resistance | 58 | 0.624 | 1.09451 |
| Q8W3E8 | A123T | Nitrogen Sensitivity | 58 | 0.19 | 0.394703 |

**S9: a)Detailed enrichment statistics for mutations near functional sites**

| **Functional Site** | **Observed Hits / Total** | **Random Hits / Total** | **% Observed** | **% Random** | **Fold Enrichment** | **p-value** | **q-value (FDR)** | **Sig. at 0.05?** |
| --- | --- | --- | --- | --- | --- | --- | --- | --- |
| PTM Sites | 3 / 16 | 1,400 / 160,000 | 18.75% | 0.875% | 21.43 | 3.46×10⁻⁴ | 5.77×10⁻⁴ | ✓ |
| Ligand Binding | 15 / 30 | 53,400 / 300,000 | 50.00% | 17.80% | 2.81 | 5.83×10⁻⁵ | 1.46×10⁻⁴ | ✓ |
| Conservation Sites | 9 / 35 | 45,700 / 350,000 | 25.71% | 13.06% | 1.97 | 3.23×10⁻² | 3.23×10⁻² | ✓ |
| Allosteric Sites | 5 / 17 | 14,700 / 170,000 | 29.41% | 8.65% | 3.40 | 1.23×10⁻² | 1.54×10⁻² | ✓ |
| Protein-Protein Interface | 25 / 89 | 64,700 / 890,000 | 28.09% | 7.27% | 3.86 | 2.85×10⁻⁹ | 1.43×10⁻⁸ | ✓ |

S9 (b) FoldX observed vs random percentage of mutations away from functional sites

| **Category** | **Definition** | **Count (N=63)** | **Percentage observed** | **Count random(N=63000)** | **Percentage random** |
| --- | --- | --- | --- | --- | --- |
| **Destabilizing** | ddG > 0.5 kcal/mol | 29 | 46.03% | 33750 | 53.57% |
| **Neutral / Mildly Stabilizing** | -0.5 kcal/mol ddG 0.5 kcal/mol | 21 | 33.33% | 11568 | 18.36% |
| **Highly Stabilizing** | ddG < -0.5 kcal/mol | 13 | 20.63% | 17682 | 28.06% |
